# Growth mechanism of oleylammonium-based tin and lead bromide perovskite nanostructures†

**DOI:** 10.1039/d4tc02029d

**Published:** 2024-08-13

**Authors:** Kushagra Gahlot, Julia N. Kraft, Manuel Pérez-Escribano, Razieh M. Koushki, Majid Ahmadi, Enrique Ortí, Bart J. Kooi, Giuseppe Portale, Joaquín Calbo, Loredana Protesescu

**Affiliations:** a Zernike Institute for Advanced Materials, University of Groningen Nijenborgh 4 Groningen 9747AG The Netherlands l.protesescu@rug.nl; b Instituto de Ciencia Molecular, Universitat de València c/Catedrático José Beltrán, 2 46980 Paterna Spain

## Abstract

Metal halide perovskites, particularly using tin and lead as bivalent cations, are well known for their synthetic versatility and ion mobility. These materials possess intriguing ionic properties that allow the formation of 2D Ruddlesden–Popper (RP) and 3D metal halide perovskite nanocrystals (NCs) under similar synthetic conditions. We studied the synthesis mechanism of oleylammonium-based Sn and Pb bromide perovskites 2D Ruddlesden–Popper (RP) in comparison with the 3D CsPbBr_3_ and CsSnBr_3_ NCs. Using experimental techniques in combination with theoretical calculations, we studied the interactions of the long-chain organic cations with the inorganic layers and between each other to assess their stability. Our findings suggest that tin bromide is more inclined toward forming higher-order RP phases or 3D NCs than lead bromide. Furthermore, we demonstrate the synthesis of precisely tuned CsSnBr_3_ 3D NCs (7 and 10 nm) using standard surface ligands. When the 3D and 2D tin halide perovskite nanostructures coexist in suspension, the obtained drop-cast thin films showed the preferential positioning of residual RP nanostructures at the interface with the substrate. This study encourages further exploration of low-dimensional hybrid materials and emphasizes the need for understanding mechanisms to develop efficient synthetic routes for high-quality tin-halide perovskite NCs.

## Abstract 2

Our experimental and theoretical studies have inferred that tin bromide is more susceptible to form higher order 2D Ruddlesden-Popper or 3D perovskite nanostructures than lead bromide.

## Graphical abstract

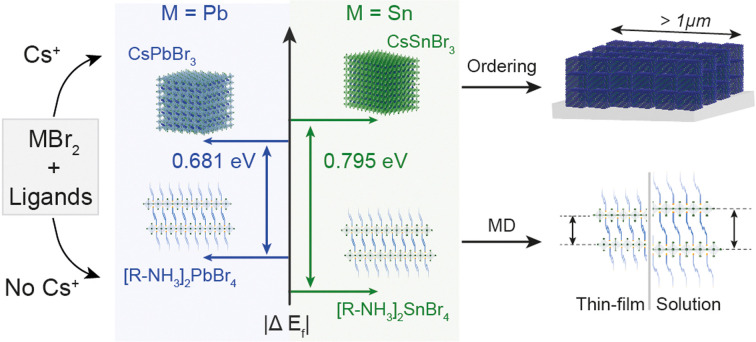

## Introduction

Tin halide perovskites are emerging as one of the desirable lead-free perovskite materials for optoelectronic applications in the form of their 3D metal halide perovskite structure (ASnX_3_), 2D Ruddlesden–Popper (RP) perovskite phases [R-NH_3_]_2_A_*n*−1_Sn_*n*_X_3*n*+1_ (*n* ≥ 1), or in the synergistic combination of both.^1–4^ The 3D perovskite structure exhibits better charge transport, whereas 2D RP phases can impart stability and defect passivation.^2,5^ Several reports in photovoltaic research have utilized the combination of 2D and 3D tin-halide perovskites to improve the performance as well as the long-term stability of the device.^6–8^

The limited number of synthetic reports on tin-halide perovskite nanocrystals (NCs) have majorly focused on iodide,^9–11^ owing to its appealing band gap in the infrared range (1.3 eV, bulk phase).^12^ On the other hand, CsSnBr_3_, the lead-free counterpart of CsPbBr_3_ NCs, has been researched primarily on bulk and thin-films for interesting phenomena like emphanisis,^13^ strong spin–orbit coupling,^14^ and for applications in optoelectronics.^15,16^ Only a few reports on the colloidal synthesis of CsSnBr_3_ NCs^17,18^ highlighted the potential of the hot-injection method when synthesizing CsSnBr_3_ and CsSnBr_1−*x*_I_*x*_ NCs. Thus, it is desirable to thoroughly investigate and optimize the synthetic process to attain high-quality Sn-halide perovskite NCs.

From our previous studies,^19^ we learned the importance of employing the sub-stoichiometric ratio of ligands (Sn : oleate : oleyl ammonium = 1 : 1 : 1) and excess of the SnI_2_ precursor to achieve stable, monodisperse, and tunable optical properties for the tin-halide perovskite NCs using a hot-injection method.^19^ The structural dynamics in the tin-halide perovskite nanostructures have been showcased with the presence of 3D CsSnI_3_ nanocuboids and 2D [R-NH_3_]_2_Cs_*n*−1_Sn_*n*_I_3*n*+1_ (*n* > 1) RP nanosheets.^19^ Directing the reaction towards a specific dimensionality in the presence of SnX_2_ precursors and an ammonium ligand (oleylammonium) has posed an arduous challenge. Density functional theory (DFT) calculations demonstrated that, for Pb-halide-based perovskites,^20^ the formation energies of these reduced dimensional structures are comparable to those of the 3D counterparts.

The synthesis of CsPbBr_3_ or CsSnI_3_ NCs, as documented in various reports,^17,19,21,22^ typically involves the solubilization of metal salts subsequent to an acid–base reaction between a carboxylate (oleate, OA, R-COO, and C_17_H_33_COO^−^) and an ammonium moiety (oleylammonium, OLA, R-NH_3_, C_18_H_35_NH_3_^+^). These processes, where the Cs^+^ cation is intercalated at a specified temperature, serve as a foundational approach for synthesizing CsSnBr_3_ NCs analogs. Given the distinct reactivities, solubilities, and complexation behaviors of PbBr_2_ and SnBr_2_, along with the potential for competitive formation of both 2D [R-NH_3_]_2_Cs_*n*−1_Sn_*n*_Br_3*n*+1_ (*n* ≥ 1) and 3D CsSnBr_3_ nanostructures, the synthesis of CsSnBr_3_ NCs remains a challenge.

In this work, we first investigated and compared the formation dynamics of oleyl ammonium-based tin- and lead-bromide 2D RP structures. We have observed that the *n* = 1 [R-NH_3_]_2_MBr_4_ 2D RP structures exhibit different interlayer *d*-spacings in solution and thin-films due to the long organic chain flexibility and the weak non-covalent interactions, with minor chain-to-chain interdigitation. Energetically, these 2D RP structures are less likely to form in the case of SnBr_2_ as compared to PbBr_2_ or SnI_2_. We continued with the synthesis of 3D CsSnBr_3_ NCs for which we optimized the reaction parameters and we isolated and investigated two different sizes with average edge lengths of ≈7 and 10 nm. The 7 nm CsSnBr_3_ NC solution showed an extended ordering up to a few microns with simple drop-casting. This research work aims to advance the understanding of dimensional dynamics in the colloidal chemistry of Sn and Pb perovskite NCs.

## Results and discussion

### Oleyl ammonium-based 2D RP perovskite structures (*n* = 1)

**Fig. 1:**
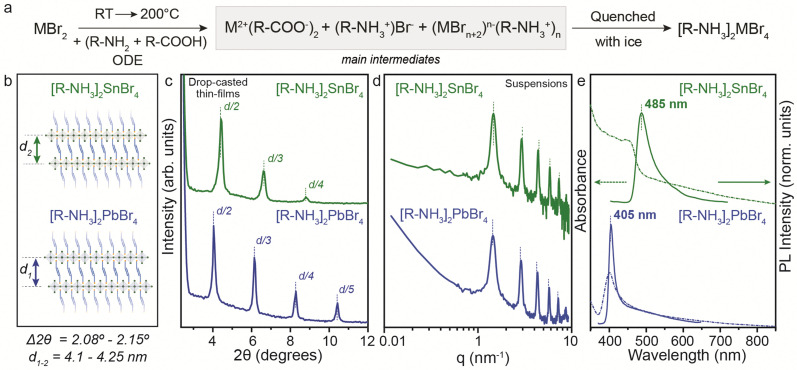
(a) General reaction scheme for the synthesis of the (*n* = 1) [R-NH_3_]_2_MBr_4_ RP perovskite phase prepared at 0.3 M. (b) Chemical structure representation of 2D [R-NH_3_]_2_MBr_4_ RP perovskites (*n* = 1). (c) The low-angle XRD pattern showcases the periodic peaks for the higher order of reflections based on an interlayer *d*-spacing ranging from 4.1 to 4.25 nm. (d) Small-angle X-ray scattering (SAXS) profiles for the 2D [R-NH_3_]_2_MBr_4_ RP perovskites (*n* = 1) suspension in toluene show periodic peaks corresponding to a *d*-spacing of 4.3 nm. (e) UV-Visible and photoluminescence spectroscopy for the 2D [R-NH_3_]_2_MBr_4_ RP perovskites (*n* = 1).

To address the synthetic challenges found in the design of low-dimensional tin-based perovskites, we first investigated the formation of 2D RP perovskite nanostructures using Sn and Pb bromide salts (SnBr_2_, PbBr_2_) as starting precursors. We already reported that SnI_2_^9,10,17,19^ and SnBr_2_^23^ can form a 2D RP perovskite phase ([R-NH_3_]_2_SnX_4_) when complexed by the OLA (R-NH_3_^+^) and OA acid–base couple in a non-coordinated solvent (here octadecene, ODE) after a heating–cooling process (Fig. 1a).^19,23^ Therefore, we used the optimized precursor ratio^19^ MX_2_ : OLA : OA = 1 : 1 : 1 at a reaction concentration of 0.3 M (Fig. 1) and 0.03 M (for Pb, see Fig. S1, ESI†). After the complete dissolution of MX_2_ at 200 °C, we quenched the reaction mixture to room temperature to precipitate the 2D RP powders: [R-NH_3_]_2_SnBr_4_ (bright yellow) and [R-NH_3_]_2_PbBr_4_ (cool-white). Note that we used the same precursor concentration as previously reported for the Sn halide perovskite synthesis (0.3 M, Fig. 1), as it corresponds to the minimum concentration that can be used to yield thermodynamically stable Sn halide perovskite NCs. Still, we also tried 0.03 M (10 times diluted), the concentration typically used for Pb halide perovskite NCs^21,22,24^ (Fig. S1, ESI†). In both cases, the 2D RP perovskite structures were further purified and characterized. The low-angle X-ray diffraction (XRD) patterns recorded for thin films of [R-NH_3_]_2_SnBr_4_ (Fig. 1c, green plots) and [R-NH_3_]_2_PbBr_4_ (Fig. 1c, blue plots) drop-cast on Si-wafer showed diffraction peaks with a *d*-spacing of 4.1 nm for [R-NH_3_]_2_SnBr_4_ and 4.25 nm for [R-NH_3_]_2_PbBr_4_. To confirm this *d*-spacing in the colloidal suspension of 2D RP perovskites, we also measured their small-angle X-ray scattering (SAXS) profile, which yields a spacing between the inorganic layers of 4.3 nm for both [R-NH_3_]_2_SnBr_4_ and [R-NH_3_]_2_PbBr_4_ RP structures (Fig. 1d).

As depicted in Fig. 1e, the UV-visible absorbance and photoluminescence (PL) measurements demonstrated pronounced quantum confinement effects for both [R-NH_3_]_2_MBr_4_ structures, featuring narrow excitonic signatures (2.74 eV for Sn and 3.1 eV for Pb) and PL peaks at 485 nm (2.56 eV) for Sn and at 405 nm (3.05 eV) for Pb, with a full width at half maximum (FWHM) of approximately 0.1 eV, in good agreement with previous reports.^25,26^ We examined the morphological characteristics of the 2D RP [R-NH_3_]_2_MX_4_ materials, revealing their presentation as nanosheets with an anticipated aspect ratio of 3 : 2, as evidenced for [R-NH_3_]_2_SnBr_4_ in Fig. S2 (ESI†).

For the PbBr_2_ salt, a similar reaction path to yield [R-NH_3_]_2_PbBr_4_ structures was observed when the reaction was performed according to the procedure reported by Protesescu *et al.*^21^ (precursor concentration ∼ 0.03 M). In this case, the formation of [R-NH_3_]_2_PbBr_4_ 2D RP perovskite structures was also confirmed. Fig. S1a (ESI†) highlights the low-angle XRD pattern with the 2*θ* periodicity corresponding to a *d*-spacing of 4.053 nm, slightly smaller than the values recorded for the concentrated system. The optical properties were not concentration-dependent, yielding similar UV-visible and PL spectra (Fig. S1b, ESI†). These 2D RP structures have low colloidal stability, so scattering effects are present in the absorption measurements.

While the chemical reaction pathway was anticipated, we were intrigued by the considerable spacing variation between the inorganic layers in those *n* = 1 structures. Similar values were reported for [R-NH_3_]_2_SnI_4_,^19,23^ which are dependent on the solid (precipitated as powder) and colloidal state, as well as on the cooling rate during the quenching of the reaction. Shorter cations were reported for both Sn and Pb 2D RP *n* = 1 structures with more rigid chains (such as butylammonium, phenylethylammonium, and octylammonium) and interspacing values ranging from 1.9 to 2.3 nm.^27,28^ Moreover, several reports described the high stability of those 2D structures with short ligands due to van der Waals interactions, making them energetically favorable over higher *n* values.^20,29,30^

**Fig. 2:**
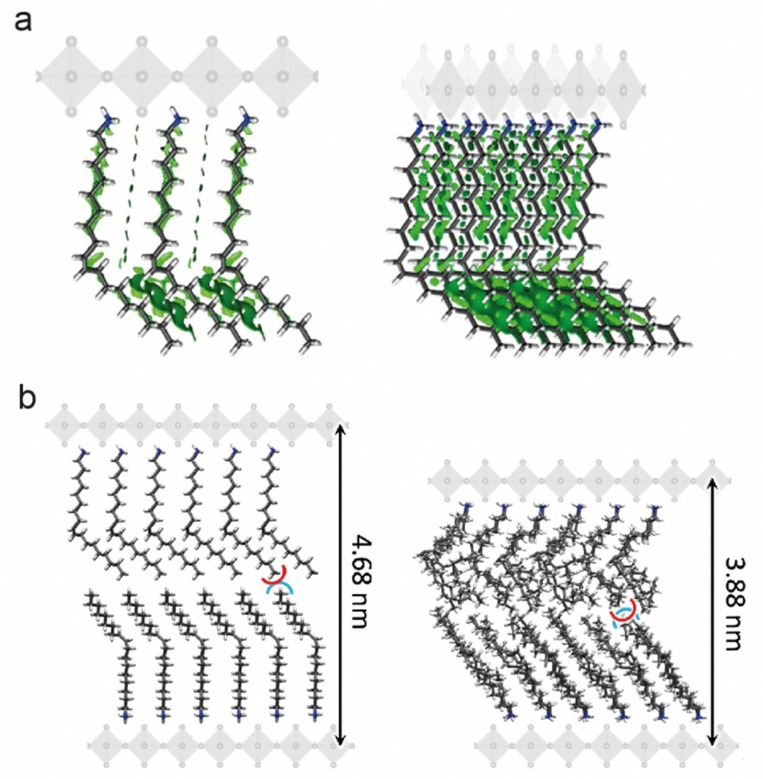
(a) Front (left) and diagonal (right) views of the NCI volumes (in green) indicating the weak but extended non-covalent forces governing the interaction between neighboring oleylammonium chains for the [R-NH_3_]_2_MBr_4_ RP perovskite (*n* = 1); rendering an iso value of 0.3 a.u. (b) *d*-spacing values calculated for the [R-NH_3_]_2_SnBr_4_ RP perovskite (*n* = 1) at the static DFT-minimum energy structure (left) and after an extended molecular dynamics simulation at room temperature (right). The location of the perovskite monolayer (not included in these calculations) is displayed for representation clarity.

The [R-NH_3_]_2_MX_4_ RP phases were investigated by means of a multilevel theoretical approach (see the ESI† for full details). First, the minimum-energy structures were calculated under the density functional theory (DFT) framework (PBEsol level of theory). Then, the extent of non-covalent interactions between the oleylammonium chains in the [R-NH_3_]_2_MX_4_ phases was estimated through analysis of the non-covalent index (NCI),^31^ as implemented in the NCIPlot code,^32^ and the interaction energy. Our calculations indicate an extended oleylammonium inter-chain region of weakly stabilizing non-covalent interactions (green surfaces in Fig. 2a). Interaction energies predicted at the PBEsol level including vdW correction suggest a slightly larger chain-to-chain stabilization in [R-NH_3_]_2_SnBr_4_ (−1.74 eV) compared to [R-NH_3_]_2_PbBr_4_ (−1.66 eV) and [R-NH_3_]_2_SnI_4_ (−1.44 eV). This trend correlates with the A-site size, and thus, with the chain-to-chain distance, which is calculated to be shorter for Sn–Br (N⋯N average distance of 6.5 Å), than for Pb–Br (6.6 Å) and Sn–I (7.0 Å). In fact, we predict negligible lattice distortions with respect to the bulk 3D analogue in [R-NH_3_]_2_SnBr_4_ compared to [R-NH_3_]_2_PbBr_4_ and [R-NH_3_]_2_SnI_4_ (see Tables S1 and S2, ESI†), indicative of optimal chain-to-chain separation in the former. On the other hand, the interaction energy between the perovskite MX_6_ octahedra and the oleylammonium cation was computed to be −5.40 eV for [R-NH_3_]_2_SnBr_4_, −5.76 eV for [R-NH_3_]_2_SnI_4_, and −6.39 eV for [R-NH_3_]_2_PbBr_4_ at the PBEsol/tier-2 level of theory. The cation–perovskite interaction is dominant compared to the chain-to-chain interaction, thus pointing to easier desorption of the oleylammonium for [R-NH_3_]_2_SnBr_4_, a process required to form higher-order 2D [R-NH_3_]_2_Cs_*n*−1_M_*n*_X_3*n*+1_ (*n* > 1) or 3D CsMX_3_ perovskite phases. The formation of 2D RP with *n* = 1 is expected to be more favorable in [R-NH_3_]_2_SnI_4_ than in the bromide analogue, which agrees with previous reports where tin-iodide 2D RP structures were synthesized and stabilized.^19^

To study the extent of intercalation between the oleylammonium chains and the effect of temperature and dynamic disorder on the interlayer *d*-spacing, classical molecular dynamics (MD) simulations were conducted on top of the DFT minimum-energy structure predicted for the [R-NH_3_]_2_SnBr_4_ phase, as a representative example. A significant contraction of the oleylammonium chains was observed along the *c*-axis when the system was equilibrated and thermalized at 298 K. The interlayer *d*-spacing decreases from 4.7 to 3.9 nm along the dynamics, with a small intercalation of <3 Å between the oleylammonium chains of neighboring layers (Fig. 2b). These results remark the flexible character of the OLA cations, and could explain the discrepancy observed in the *d*-spacings for drop-cast thin films and suspensions of [R-NH_3_]_2_MX_4_ RP structures. There is expected to be a decrease in the *d*-spacing variability on shortening the carbon chain length and rigidifying the structure of the organic cation.

### Mechanistic studies for CsMBr_3_ NCs (M = Pb and Sn)

**Fig. 3:**
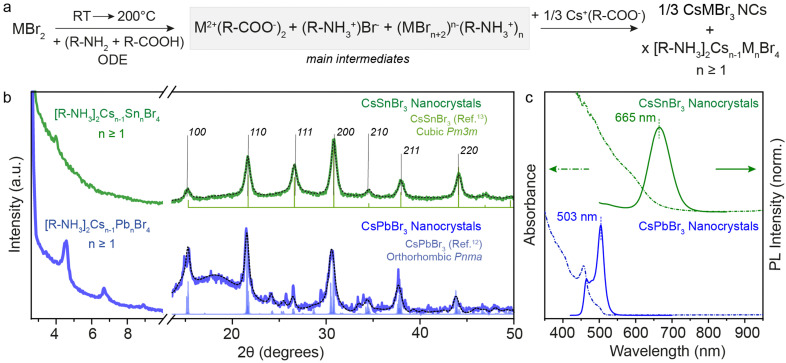
(a) General reaction scheme for the metal bromide case when Cs(Ol) is injected. (b) XRD pattern of 3D CsSnBr_3_ and CsPbBr_3_ NCs showcasing the residual presence of 2D [R-NH_3_]_2_Cs_*n*−1_Sn_*n*_Br_3*n*+1_ and [R-NH_3_]_2_Cs_*n*−1_Pb_*n*_Br_3*n*+1_ with *n* ≥ 1. (c) UV-Visible and PL spectroscopy of 3D CsSnBr_3_ and CsPbBr_3_ NCs showing red-shift from their respective *n* = 1 2D [R-NH_3_]_2_MX_4_ RD perovskites shown in Fig. 1. The black dotted line plots the Rietveld refined XRD data for respective crystal phases.

To synthesize CsSnBr_3_ NCs starting with the same precursor composition as for the 2D RP structures discussed above, we utilized the hot-injection of the Cs(oleate) precursor into our main intermediates at 200 °C followed by subsequent quenching with ice-water. Fig. 3a shows the structural and optical characterization of the obtained CsSnBr_3_ NCs in comparison to CsPbBr_3_ NCs. Note that for this comparison, we used the 0.3 M concentration. Fig. S3 (ESI†) displays the Sn-halide salt concentration-dependent optical stability of NCs, which shows the necessity of performing Sn-halide perovskite NC synthesis at supersaturated concentrations (>0.3 M) for longer stability of NCs.^19^ Thus, to make long-standing, colloidally stable NCs, the synthesis of Sn-halide perovskite NCs must be performed at a higher concentration of Sn while maintaining the sub-stoichiometric ratio of ligands (SnX_2_ : OLA : OA = 1 : 1 : 1) with respect to the Sn salt.^33^

The XRD patterns recorded for both metal bromide perovskite cases are shown in Fig. 3b. The presence of sharp periodic peaks at low 2*θ* angles confirms the formation of 2D RP perovskite structures, which may have discrete intercalation of Cs cations giving rise to different order of 2D structures. To study the relative stability of 2D RP [R-NH_3_]_2_Cs_*n*−1_M_*n*_X_3*n*+1_ structures with *n* > 1, DFT structural optimizations and formation energy estimations were conducted at the PBEsol level for M = Pb/Sn and X = Br, and *n* = 1–4. The values predicted for the formation energies, listed in Table S4 (ESI†), align well with the XRD findings since all high-order [R-NH_3_]_2_Cs_*n*−1_M_*n*_X_3*n*+1_ phases (*n* > 1) are stable and close in energy. However, at wider angles, the 3D crystal phase was observed with high FWHM, suggesting the formation of 3D NCs. CsSnBr_3_ (green) and CsPbBr_3_ (blue) NCs crystallize in the purely cubic (*Pm*3*m*) and orthorhombic (*Pnma*) crystal phase, respectively, as measured with the lab-scale powder X-ray diffractometer and compared with the bulk.^12,13,34^ For completeness, Fig. S4 (ESI†) shows the XRD pattern of CsSnI_3_ NCs at low and high diffraction angles, which is similar to that of CsPbBr_3_ with periodic 2*θ* peaks and orthorhombic (*Pnma*) crystal phase. When comparing these results for OA and OLA acid–base couple reactions and with a high concentration of salts (0.3 M), a nearly complete conversion of precursors to 3D NCs was observed in the case of CsSnBr_3_, in contrast to what is obtained for CsPbBr_3_ or CsSnI_3_ perovskite compositions. This observation can be rationalized by considering the smaller binding energy between the cation and the perovskite calculated for the 2D [R-NH_3_]_2_SnBr_4_ phase (as described above), together with a smaller formation energy per formula unit (*E*_f_ = −0.175 eV) compared to [R-NH_3_]_2_PbBr_4_ (*E*_f_ = −0.247 eV) and [R-NH_3_]_2_SnI_4_ (*E*_f_ = −0.325 eV). However, upon increasing *n*, the formation energy rapidly becomes more favorable, especially for the tin-bromide composition (*n* ≥ 3, Tables S3 and S4, ESI†). The net stabilization between the 2D [R-NH_3_]_2_MX_4_ phase and that for bulk, which for comparison is assumed to be that of *n* = 4, is calculated larger for [R-NH_3_]_2_SnBr_4_ (0.79 eV) than for [R-NH_3_]_2_PbBr_4_ (0.68 eV) and [R-NH_3_]_2_SnI_4_ (0.50 eV). The formation of CsSnBr_3_ NCs is therefore highly likely to be in a more phase-pure tin-bromide perovskite 3D structure, compared to the other metal halide perovskite NCs, which agrees with that observed experimentally.

Fig. 3c exemplifies the quantum confinement effect through absorbance and PL spectra of 3D CsSnBr_3_ and CsPbBr_3_ NCs with the residual presence of [R-NH_3_]_2_Cs_*n*−1_Sn_*n*_Br_3*n*+1_ and 2D [R-NH_3_]_2_Cs_*n*−1_Pb_*n*_Br_3*n*+1_ with *n* ≥ 1. Cuboidal 10 nm 3D CsSnBr_3_ NCs showed a broad excitonic peak (600 nm) and a PL peak centered at 665 nm (FWHM ∼ 0.18 eV), whereas narrower excitonic peaks were observed for orthorhombic 3D CsPbBr_3_ NCs (458 and 500 nm, respectively) with main emission peak at 503 nm (FWHM ∼ 0.12 eV).

In order to further explore and rationalize the synthesis of 3D Sn and Pb halide perovskites NCs in the absence of 2D RP structures, one could decrease the oleylammonium cation concentration or utilize a weaker and sterically hindered protic acid. Thus, we used diisooctylphosphinic acid (DOPA) as an acid and surfactant for this reaction instead of OA. When the MX_2_ salts were dissolved in the presence of the DOPA and OLA couple, the formation of clean 2D [R-NH_3_]_2_PbBr_4_, [R-NH_3_]_2_SnBr_4_, and [R-NH_3_]_2_SnI_4_ 2D RP perovskite structures was observed (Fig. S5a, ESI†). It is assumed that the reaction intermediates are potentially the same as in the case of the OA/OLA couple. We then proceeded with the addition of the Cs cation as Cs-DOPA. For PbBr_2_, this addition leads to the formation of stable phase-pure orthorhombic CsPbBr_3_ NCs as characterized *via* XRD pattern and presented in Fig. S5b (ESI†). In stark contrast, the Sn-halide perovskite NCs degraded very quickly during the cooling process, and no CsSnBr_3_ or CsSnI_3_ NCs could be identified. The degradation products of these reactions, when analyzed, and the XRD patterns showed the presence of mainly 2D RP [R-NH_3_]_2_Cs_*n*−1_Sn_*n*_Br_3*n*+1_ structures (*n* ≥ 1) for SnBr_2_ and a mixture of 2D RP [R-NH_3_]_2_Cs_*n*−1_Sn_*n*_I_3*n*+1_ structures (*n* ≥ 1) and CsI (cubic, *Pm*3*m*) for SnI_2_ (Fig. S5b, ESI†). Our results thus prove that DOPA, being a weakly binding ligand, is unable to stabilize the 3D perovskite structure for the Sn-halide perovskite NCs but can do so for Pb-halide perovskite NCs in the presence of OLA. For Sn-halide perovskite NCs, we have observed that in the presence of only DOPA, uncontrolled nucleation and growth yields bulk 3D Sn-halide perovskites (Fig. S6, ESI†).

### Structural, morphological and optical characterization of CsSnBr_3_ NCs

Thus far, our understanding has been advanced through both experimental and theoretical investigations into the reactivity of precursors and the mechanisms dictating the transformation from 2D RP structures to 3D CsSnBr_3_ NCs, particularly in comparison to analogous lead-based compounds and other halides. In light of this consideration, CsSnBr_3_ NCs were synthesized and their distinctive characteristics are further discussed below.

**Fig. 4:**
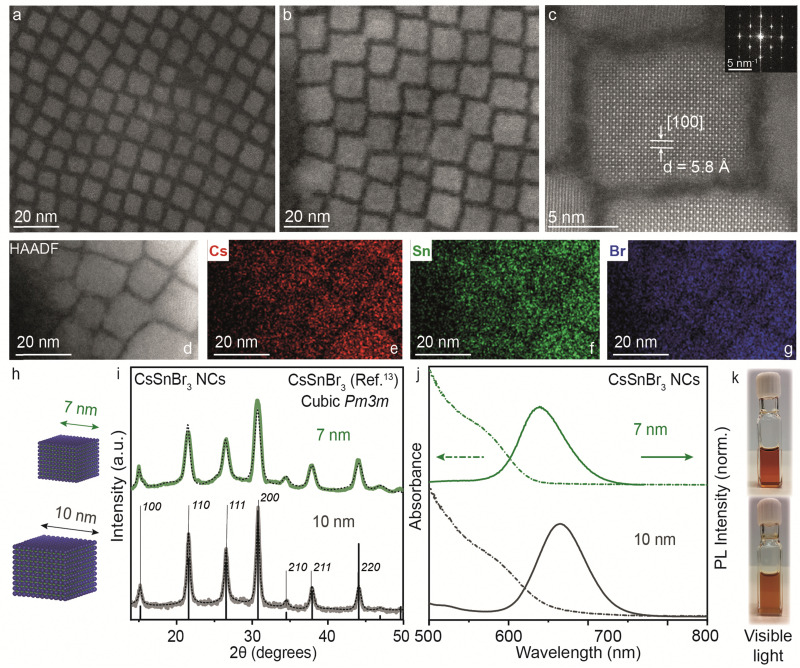
(a) and (b) STEM images of 7 and 10 nm CsSnBr_3_ NCs, respectively. (c) High-resolution STEM showing lattice parameter of 5.8 Å corresponding to the [100] planes for a 10 nm CsSnBr_3_ NC. Inset: Cubic FFT pattern for the CsSnBr_3_ NC shown in (c). (d)–(g) STEM-energy dispersive X-ray (EDX) elemental mapping for the image area depicted in (d) with the distribution of Cs (red) (e), Sn (green) (f), and Br (blue) (g). (h) Schematic of CsSnBr_3_ NCs with average sizes of 7 and 10 nm. (i) XRD pattern of CsSnBr_3_ NCs with an average size of 7 (green) and 10 nm (dark grey) plotted with cubic *Pm*3*m* bulk reference (black). (j) UV-Visible and steady-state PL spectra of 7 (green) and 10 nm (dark grey) CsSnBr_3_ NCs. (k) Photographs of 7 (top panel) and 10 nm (bottom panel) CsSnBr_3_ colloidal NCs dispersed in toluene in visible light. The black dotted line plots the Rietveld refined XRD data for respective crystal phases.

In the bulk, CsSnBr_3_ exists in the symmetrical cubic *Pm*3*m* crystal phase at room temperature with a band gap of 1.75 eV (∼700 nm). This crystal phase undergoes a transformation to a tetragonal *P*4/*mbm* phase around 286 K, which then shows a continuous transition to an orthorhombic *Pnma* phase around 250 K.^13^ Our synthesized CsSnBr_3_ NCs also showed *Pm*3*m* crystal phase, highly monodisperse NCs in cuboidal shape with an average edge length of 7 and 10 nm as shown in Fig. 4. Fig. S7a–d (ESI†) provides the scanning transmission electron microscopy (STEM) images at different magnifications for 7 and 10 nm CsSnBr_3_ NCs, corroborating their monodispersity with size histograms (Fig. S7e and f, ESI†). This crystal phase is further confirmed with the high-resolution STEM (HR-STEM) analysis, demonstrating a *d*-spacing of 5.8 Å corresponding to the [100] planes, see also the fast-Fourier transform (FFT) pattern presented in the inset of Fig. 4c. STEM energy dispersive X-ray spectroscopy (EDXS) elemental mapping performed on the 10 nm CsSnBr_3_ NCs sample shows an even distribution of Cs, Sn, and Br over the NCs with atomic percentages in the ratio 1 : 1.3 : 3, respectively, establishing the correct stoichiometry of the NC system (Fig. 4d–g). Table S1 (ESI†) tabulates the elemental analysis results acquired by STEM-EDXS and inductively coupled plasma-mass spectrometry (ICP-MS) for 7 and 10 nm CsSnBr_3_ NCs. The XRD pattern measured with lab instrumentation shown in Fig. 4i exhibits CsSnBr_3_ cubic *Pm*3*m* perovskite crystal phase with the apparent change in the FWHM for the two different sizes of CsSnBr_3_ NCs. UV-Visible and steady-state PL spectra for different sizes are plotted in Fig. 4j, showing the emission peaks at 638 nm for 7 nm (FWHM = 0.18 eV) and at 665 nm for 10 nm (FWHM = 0.19 eV) CsSnBr_3_ NCs with the visible light photographs of colloidal NCs suspensions (Fig. 4k). To confirm the interaction of ligands with perovskite NC surface, we performed the Fourier transform-infrared (FT-IR) spectroscopy showing the disappearance C=O stretching frequency (≈1700 cm^−1^) in NCs which is present in the free oleic acid ligand (Fig. S8, ESI†).

We further explored the variation in the synthetic parameters. For the lower (Cs : Sn < 1 : 3) and higher cation (Cs : Sn > 1 : 3) ratios, the mixture of 2D [R-NH_3_]_2_Cs_*n*−1_Sn_*n*_Br_3*n*+1_ RP perovskite and 3D CsSnBr_3_ NCs was obtained (Fig. S9, ESI†), with an extra excitonic peak observed at 526 nm (2.36 eV) with emission at 540 nm (FWHM = 0.19 eV) for Cs : Sn = 1 : 6, and a broadening of the excitonic peak with a shoulder emission towards lower wavelength at 542 nm (FWHM = 0.18 eV) for Cs : Sn = 1 : 2. Thus, the cation ratio of Cs : Sn = 1 : 3 seems ideal, which also correlates well with our previous report on CsSnI_3_ NCs.^19^ Furthermore, the reactions performed at lower temperatures (from 100 to 160 °C) lead to the preferential formation of 2D RP structures rather than 3D perovskites. As discussed above, DFT calculations predict that the formation of 3D metal halide perovskites is energetically favored compared to 2D RP perovskite structures, leading to the formation and stabilization of the 3D perovskite structure. When probing various kinetic parameters, the injection temperature has a notable effect on the formation of the 3D or 2D RP structures. Fig. S10a (ESI†) shows the XRD pattern of the CsSnBr_3_ NCs synthesized at 100 °C in comparison with those formed at 200 °C. At the low angles, sharp periodic peaks are observed with *d*-spacing of ∼4.4 nm, which suggests the formation of higher order *n* > 1 [R-NH_3_]_2_Cs_*n*−1_Sn_*n*_Br_3*n*+1_ RP structures in combination with 3D perovskite NCs. The blue shift in the absorbance and PL peak maxima shown in Fig. S10b (ESI†) confirms the confinement effects arising from the dimensionality reduction. Fig. S11 (ESI†) shows scanning electron microscopy (SEM) images in transmission mode for the reaction performed at 100 °C, indicating the co-existence of 3D CsSnBr_3_ NCs and 2D nanosheets. Since it is difficult to stabilize the discrete higher-order *n* > 1 [R-NH_3_]_2_Cs_*n*−1_Sn_*n*_Br_3*n*+1_ RP perovskites, it is not possible to assign the *n* precisely. It is also clear from the literature that as *n* becomes higher, the isolation and stabilization of the RP structure becomes equally difficult.^20^ These observations suggest that at low temperatures, the crystallization of 2D RP structures is preferred rather than 3D perovskites, and depending on the concentration of the inorganic cation (Cs^+^), intercalation (value of *n*) can vary to a different extent.

The change in the reaction medium to a more volatile, non-polar, non-coordinating solvent (*i.e.* mesitylene) was performed to disrupt the ligand–solvent interactions and facilitate the post-synthetic treatment of the NCs. The reactions with mesitylene were performed under similar precursor ratios but at lower temperatures (from 70 to 150 °C), which resulted in products containing 3D CsSnBr_3_ NCs with PL emission ranging from 603 to 652 nm (Fig. S12, ESI†). Upon lowering the temperature (<150 °C), 2D [R-NH_3_]_2_Cs_*n*−1_Sn_*n*_Br_3*n*+1_ RP perovskites were obtained together with 3D CsSnBr_3_ NCs (Fig. S12, ESI†), and a blue shift of the optical characteristics with excitonic peak at 555 nm and an emission peak at 605 nm was observed. The change in solvent also corroborates the fact that the reaction performed at lower temperatures (≤150 °C) is likely to form 2D RP structures rather than 3D NCs. Fig. S13 (ESI†) compares the optical properties of the 2D and 3D tin-halide perovskite materials.

### CsSnBr_3_ NCs thin films

**Fig. 5:**
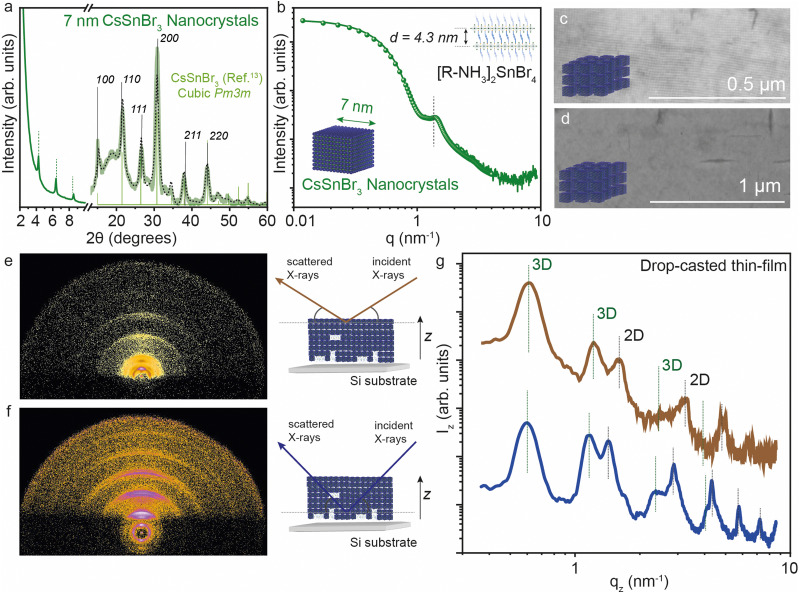
(a) XRD of 7 nm CsSnBr_3_ NCs. (b) Solution SAXS profile of 7 nm CsSnBr_3_ NCs together with the best fit using a mixed ensemble of spheroidal particles and 2D colloidal crystallites. (c) and (d) SEM images of 7 nm CsSnBr_3_ NCs showing extended ordering characteristics. (e) and (f) GIXS images on a drop-cast thin-film of 7 nm CsSnBr_3_ NCs and 2D [R-NH_3_]_2_SnBr_4_*n* = 1 RP structures at the low (∼10 nm) and high (∼200 nm) penetration depth, respectively, together the schematic representation. (g) GIXS vertical profiles on drop-cast thin-film of 7 nm CsSnBr_3_ NCs at the low (∼10 nm) and high (∼200 nm) depth, respectively. The black dotted line plots the Rietveld refined XRD data for respective crystal phases.

We further analyzed the self-assembly behavior of the CsSnBr_3_ NCs and the impact of the 2D [R-NH_3_]_2_Cs_*n*−1_Sn_*n*_Br_3*n*+1_ RP structures on the in-plane and out-of-plane extended ordering. We selected the smaller NCs for this since we expected the co-presence of some 2D crystallites, as visible in XRD (Fig. 5a). The average size of the size-selected CsSnBr_3_ NCs was investigated by suspension SAXS, which shows a dominant diffuse intensity modulation generated by the presence of particles with 7 ± 1 nm diameter, in close agreement with the TEM results, together with a minor fraction of the 2D crystallites (Fig. 5b). The nanocubes show a remarkable tendency to form long-ranged ordered structures, namely superlattices, which can be easily obtained using a facile drop-cast thin-film technique (concentration of NCs ∼ 20 mg mL^−1^). These superlattices show order above 1 μm when imaged by SEM (Fig. 5c and d). To probe the superlattice formation at a large length scale, grazing incidence X-ray scattering (GIXS) patterns were acquired, illuminating mm^2^ of the thin NCs films deposited on a silicon substrate. The GIXS patterns reveal the co-assembly of the cubic NCs and the layered 2D crystallites, both showing the degree of particle alignment (Fig. 5e and f).

To learn about the vertical distribution of the co-assembled 3D CsSnBr_3_ NCs and 2D [R-NH_3_]_2_SnBr_4_*n* = 1 RP structure, GIXS measurements at two different incident angles (0.2° and 1.8°). Have been performed, probing the films at different depths (∼10 nm and ∼200 nm, respectively) Fig. 5e and f show the GIXS vertical profiles for a drop-cast film using the suspension of 7 nm CsSnBr_3_ NCs at the ∼10 and ∼200 nm depths, respectively. As marked in the figure, both profiles show evidence of the 3D and 2D superlattices. For the 3D superstructure, the first peak is the [100] peak for the 3D cubic superstructure located at 0.6 nm^−1^ which is a lattice dimension of 9 nm, in line with the 7 nm NC dimension and 2 nm of organic ligand. For the 2D phase, the [001] peak gives a *d*-spacing of ∼4.3 nm, in agreement with that observed above in Fig. 1 by XRD and SAXS. By comparing the profiles obtained from the GIXS patterns acquired at the two different incident angles, the presence of 2D [R-NH_3_]_2_SnBr_4_ can be observed more at a higher X-ray penetration depth than near the surface, suggesting that 2D RP crystallites are preferentially present at the bulk of the thin film rather than on the surface.

## Conclusions

In summary, our extensive investigation has provided significant insights into the synthesis, characterization, and properties of 2D Ruddlesden–Popper (RP) perovskite structures using tin (Sn) and lead (Pb) bromide salts as starting materials. Building upon prior research, we have effectively synthesized and refined 2D RP [R-NH_3_]_2_SnBr_4_ and [R-NH_3_]_2_PbBr_4_ structures employing controlled heating and cooling techniques, in conjunction with complexation involving the OLA and OA acid–base couple within a non-coordinated solvent (ODE). Our analysis has unveiled pronounced quantum confinement effects in both *n* = 1 structures, as corroborated by UV-visible absorbance and PL measurements. Morphological examinations have substantiated the formation of nanosheets displaying anticipated aspect ratios, indicative of their inherent 2D nature.

Leveraging DFT calculations and MD simulations, we have elucidated the underlying mechanisms dictating the formation and behavior of 2D RP perovskite structures. Our simulations have provided valuable insights into the influence of temperature, dynamic ordering, and intermolecular interactions on the observed variations in *d*-spacing values within these structures. A comprehensive investigation into CsSnBr_3_ NCs and their thin film processing has further clarified the assembly behavior, demonstrating the capabilities of co-assembly to form long-range ordered superstructures.

Our findings significantly contribute to elucidating synthetic methodologies for engineering metal-halide perovskite nanostructures with tailored properties. Moreover, we provide a framework for investigating other novel materials such as germanium, bismuth halides, and metal-chalcogenide perovskite nanostructures, thus advancing the frontiers of materials science research.

## Experimental

### Materials

Cesium carbonate (Sigma, metals basis, 99.9%), tin(ii) bromide (TCI Europe, 99%), tin(ii) iodide (Sigma, anhydrous beads, 99.99%), octadecene (ODE, Sigma, tech. grade, 90%), oleic acid (OA, Sigma, tech. grade, 90%), oleyl amine (OLA, Sigma, tech. grade, 70%), diisoctylphosphinic acid (DOPA, Sigma, 90%), and toluene (Acros, extra dry, 99.85%) were used.

### Synthesis of Cs-precursors

#### Cesium oleate (0.222 M)

Cs_2_CO_3_ (1.630 g, 5 mmol), OA (5 ml, 16 mmol), and ODE (40 ml) were loaded into a 100 ml 3-neck round-bottom flask and vigorously stirred under vacuum for one hour at 120 °C. Additionally, the reaction mixture was heated up to 150 °C under a N_2_ flow to ensure the complete conversion of cesium carbonate into cesium oleate. The obtained cesium oleate solution was stored under an N_2_ atmosphere and heated up to 100 °C before being used.

#### Cesium diisooctyl phosphinate (0.25 M)

Cs-DOPA was prepared using a modified procedure from Shynkarenko *et al.*^35^ Cesium carbonate (0.40 g, 1.25 mmol), DOPA (2 mL, 6.4 mmol), and ODE (8 ml) were loaded into a 25 ml 3-neck round-bottom flask and vigorously stirred under vacuum for one hour at 120 °C. Under N_2_ flow, the temperature of the reaction mixture was increased until it becomes transparent (~140 °C). The solution was then cooled down and stored at room temperature under an inert atmosphere (N_2_).

### Synthesis of 2D RP structures [R-NH_3_]_2_MX_4_ (R-oleyl)

2 mmol of MX_2_, (SnBr_2_ – 0.558 g; PbBr_2_ – 0.73 g; SnI_2_ – 0.75 g) along with 2 mmol of dried acids (OA – 0.63 mL; DOPA – 0.63 mL), 2 mmol of OLA (0.66 mL), and ODE (5 mL) were loaded into a 25 mL 3-neck flask inside a N_2_ glovebox. The reaction mixture was carefully transferred to a Schlenk line and stirred under vacuum for 10 minutes at room temperature, after which the temperature was increased to 105 °C and vigorously stirred for 45 minutes. The temperature was further increased to 200 °C under a N_2_ flow, the reaction was then quenched to room temperature using an ice-water bath after 5 seconds. The reaction flask was carefully transferred to the glovebox for the purification step. The crude solution was equally divided into two centrifuge tubes and centrifuged at 5000 rpm (7081 rcf) for 3 minutes. The supernatant was discarded, and the precipitate was re-dispersed in toluene (5 mL) followed by centrifugation at 13 000 rpm (18 412 rcf) for 5 minutes. The supernatant was discarded and the colloidally unstable yellow precipitate was stored in 5 mL toluene in the glovebox for further measurements.

### Synthesis of CsSnBr_3_ NCs using OA and OLA

SnBr_2_ (2 mmol, 0.558 g) along with dried oleic acid (OA, 0.63 mL, 2 mmol), oleylamine (OLA, 0.66 mL, 2 mmol), and octadecene (ODE, 5 mL) were loaded into a 25 mL 3-neck flask inside a N_2_ glovebox. The reaction mixture was carefully transferred to a Schlenk line and stirred under vacuum for 10 minutes at room temperature, after which the temperature was increased to 105 °C and vigorously stirred for 45 minutes. The temperature was further increased to 200 °C under a N_2_ flow, and 2.8 mL (0.622 mmol) cesium oleate (0.222 M) was swiftly injected. The reaction was stopped after 5 seconds by quickly immersing the reaction flask into an ice-water bath. The reaction flask was carefully transferred to the glovebox for the purification step. The crude solution was equally divided into two centrifuge tubes. Size selection for the 7 nm CsSnBr_3_ NCs: reaction mixture was centrifuged at 13 000 rpm (18 412 rcf) for 5 minutes. The supernatant was discarded, and the precipitate was re-dispersed in toluene (5 mL) followed by centrifugation at 3000 rpm (4248 rcf) for 3 minutes. The supernatant was separated out carefully and stored in the glovebox for further measurements. The precipitate was discarded. The supernatant can be further centrifuged the following day at 3000 rpm (4248 rcf) for 3 minutes to attain superior monodispersity. This supernatant was used for making superlattices. Size selection for the 10 nm CsSnBr_3_ NCs: reaction mixture was centrifuged at 5000 rpm (7081 rcf) for 3 minutes. The supernatant was discarded, and the precipitate was re-dispersed in toluene (5 mL) followed by centrifugation at 13 000 rpm (18 412 rcf) for 5 minutes. The supernatant was discarded and the precipitate was re-dispersed in 5 mL toluene, and stored in the glovebox for further measurements.

### Synthesis of CsMX_3_ NCs using DOPA and OLA

2 mmol of MX_2_ (SnBr_2_ – 0.558 g; PbBr_2_ – 0.73 g; SnI_2_ – 0.75 g), 2 mmol of dried acids (OA – 0.63 mL; DOPA – 0.63 mL), 2 mmol of OLA (0.66 mL), and ODE (5 mL) were loaded into a 25 mL 3-neck flask inside a N_2_ glovebox. The reaction mixture was carefully transferred to a Schlenk line and stirred under vacuum for 10 minutes at room temperature, after which the temperature was increased to 105 °C and vigorously stirred for 45 minutes. The temperature was further increased to 200 °C under a N_2_ flow, and Cs-DOPA (2.8 mL, 0.67 mmol) was quickly injected. The reaction was stopped after 5 seconds by quickly immersing the reaction flask into an ice-water bath. The reaction flask was carefully transferred to the glovebox for the purification step. The crude solution was equally divided into two centrifuge tubes and centrifuged at 13 000 rpm (18 412 rcf) for 5 minutes. For SnBr_2_ and SnI_2_, the precipitate turns pale white in colour from dark brown while the supernatant becomes transparent in the washing process. For PbBr_2_, the supernatant was discarded, and the precipitate was re-dispersed in toluene (5 mL) followed by centrifugation at 3000 rpm (4248 rcf) for 3 minutes. The supernatant was separated out carefully and stored in the glovebox for further measurements.

### Synthesis of CsMX_3_ NCs using only DOPA

2 mmol of MX_2_ (SnBr_2_ – 0.558 g; PbBr_2_ – 0.73 g; SnI_2_ – 0.75 g), were mixed with dried DOPA (0.63 mL, 2 mmol), and ODE (5 mL) were loaded into a 25 mL 3-neck flask inside a N_2_ glovebox. The reaction mixture was carefully transferred to a Schlenk line and stirred under vacuum for 10 minutes at room temperature, after which the temperature was increased to 105 °C and vigorously stirred for 45 minutes. The temperature was further increased to 200 °C under a N_2_ flow, the dissolution of SnBr_2_ and SnI_2_ salts was observed while PbBr_2_ remains insoluble. Without the Cs cation, the reaction was quenched to room temperature using an ice-water bath after 10 seconds to obtain a complexation product. With the Cs cation, a Cs-DOPA (2.8 mL, 0.67 mmol) was swiftly injected which led to dark brown/black color solution in the case of SnBr_2_ and SnI_2_, the reaction is then quenched to room temperature using an ice-water bath after 10 seconds. The reaction flask was carefully transferred to the glovebox for the purification step. The crude solution was equally divided into two centrifuge tubes and centrifuged at 5000 rpm (7081 rcf) for 3 minutes. The supernatant was discarded, and the precipitate was re-dispersed in toluene (5 mL) followed by centrifugation at 13 000 rpm (18 412 rcf) for 5 minutes. The supernatant was discarded and the colloidally unstable white precipitate was stored in 5 mL of toluene in the glovebox for further measurements.

### Dropcasted thin-film of 7 nm CsSnBr_3_ NCs

10 mm × 10 mm soda-lime glass substrates were cleaned with soap water and washed with distilled water. The substrates were then subjected to sonication for 15 min in ethanol, dried, and sonicated for another 15 min in acetone, followed by drying under a strong airflow. The cleaned substrates were transferred to the glove box. 20 μL of 7 nm CsSnBr_3_ NC solution with a concentration of ≈25 mg ml^−1^ was drop-cast on the glass substrate and kept under vacuum in the antechamber of the glove box for 15 minutes. This drop-cast thin-film was utilized for further characterization.

### Characterization

#### XRD

For sample preparation, a concentrated solution of the sample was drop-cast on a Si-wafer in an inert dome sample holder and the measurement was performed on a Bruker D8 Advanced diffractometer aligned in Bragg–Brentano geometry using Cu Kα radiation (*λ* = 1.54 Å) and a Lynxeye detector.

#### STEM

The samples were prepared on an ultrathin grid with 400 mesh, Cu (Ted Pella, Inc. 01822-F) which was wrapped with graphene on one side. The sample was then drop-cast on the graphene side of the grid which was then sandwiched between two graphene layers using the other grid. The TEM grid was dried overnight in the antechamber of the glove box. The measurements were performed on a Thermo Fisher Themis Z STEM operating at 300 kV.

#### SEM

The sample was prepared by drop-casting dilute sample solution onto an ultrathin grid with 400 mesh, Cu (Ted Pella, Inc. 01822-F). The measurements were performed using a FEI Helios G4 CX electron microscope in the scanning transmission mode operated at 18 kV.

#### Steady-state PL spectroscopy

Samples were prepared in a quartz cuvette loaded and sealed in the glove-box with samples dissolved in toluene (O.D. ∼ 0.2–0.8). The measurements were performed on a Horiba Scientific Jobin Yvon spectrometer equipped with a PMT detector.

#### UV-Visible absorbance spectroscopy

Samples were prepared in a quartz cuvette loaded and sealed in the glove-box with samples dissolved in toluene (O.D. ∼ 0.2–0.8). The measurements were performed on a table-top Avantes UV-Vis spectrophotometer using a tungsten and halogen filament lamp as the excitation source.

#### SAXS/GIXS

Solution SAXS experiments have been performed at the multipurpose X-ray instrument for nanostructural characterization (MINA) at the University of Groningen. The instrument is built on a high-intensity Cu rotating anode X-ray source, providing a parallel collimated X-ray beam with a photon wavelength of *λ* = 0.1543 nm. The scattering patterns were collected using a 2D Vantec detector from Bruker. In order to explore a very broad *q*-range (0.05–8 nm^−1^, where *q* is the modulus of the scattering vector 1 = 4π sin *θ*/*λ*), the SAXS data were acquired using two different sample-to-detector distances of 3 m and 0.24 m. The colloidal suspensions were contained in a quartz capillary of 1.5 mm outer diameter with 0.01 mm wall thickness. After subtracting the scattering signal from the solvent background and radial integration from 2D patterns to 2D intensity profiles, the two data sets were merged to generate the final *I*(*q*) *vs. q* SAXS curve. The sample-to-detector distance and the beam center position were calibrated using the scattered rings from a standard silver behenate powder sample. The sample with ∼5 mg mL^−1^ concentration was employed for the measurements loaded and sealed in the capillary in the glove box. The SAXS profile reported in Fig. 5b for the CsSnBr_3_ NC solution has been modeled using a curve composed of the sum of two components. One describing the scattering of the nanocubes, using the analytical expression for an ensemble of diluted spherical particles with a Gaussian distribution *D*(*R*) of the particle with average radius *R*
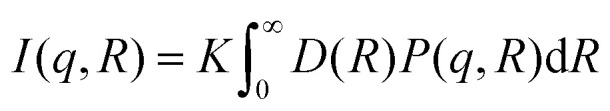
where *K* is a scaling constant (depending on the incoming beam flux, the particle concentration and the particle contrast), *P*(*q*,*R*) is the form factor for a spherical object.^3,4^ The assumption of spherical shape is plausible here, as in solution the cubes have all possible orientations and resample to spherical objects. The best fit gave NC dimensions of 7 ± 1 nm. The scattering from the colloidal 2D RP crystallites has been modelled using the expression for thin 2D slabs with a certain lateral dimension *L* and thickness *t*, vertically stacked at a distance *d*.^36,37^ The best fit gave a value of *d* = 4.3 nm, *L* = 10 nm and *t* = 1 nm. An average of *N* = 6 stacked slabs were obtained.

#### DFT calculations

Periodic boundary conditions were imposed and tier-2 numerical atom-centered orbitals (NAO) basis functions were used in conjunction with the GGA PBEsol functional,^38^ as implemented in the FHI-aims code.^39^ Relativistic effects were considered through the use of the scalar ZORA scheme. The initial structures for the bulk CsMX_3_ phases were obtained from the Materials Project database,^40^ considering γ-orthorhombic phases for CsPbBr_3_ and CsSnI_3_ (code references mp-567629 and mp-568570, respectively) and the α-cubic phase for CsSnBr_3_ (code reference mp-27214). Further details can be found in the ESI.†

## Author contributions

The manuscript was written through the contributions of all authors. All authors have given approval to the final version of the manuscript. KG performed the experiments and analysis and wrote the manuscript. JNK performed the experiments and analysis. MPE, EO and JC performed the DFT calculations and reviewed and edited the manuscript. RMK & GP performed the SAXS/GIWAXS measurements and analysis. MA and BJK performed the electron microscopy measurements. LP designed and supervised the full project and wrote the manuscript.

## Data availability

The data supporting this article have been included as part of the ESI.†

## Conflicts of interest

There are no conflicts to declare.

## Supplementary Material

TC-012-D4TC02029D-s001
