# Supplementary material for: Growth mechanism of oleylammonium-based tin and lead bromide perovskite nanostructures
Source: J Mater Chem C Mater. 2024 Aug 13;12(37):15152–62. doi: 10.1039/d4tc02029d (PMC11367222; doi:10.1039/d4tc02029d)
Supplement: TC-012-D4TC02029D-s001 [file TC-012-D4TC02029D-s001.pdf]

Supplementary Information (SI) for Journal of Materials Chemistry C.
This journal is © The Royal Society of Chemistry 2024

Supporting Information

Growth Mechanism of Oleylammonium-Based Tin and Lead Bromide Perovskite Nanostructures

Kushagra Gahlot [a], *Julia N. Kraft* [a], *Manuel Pérez-Escribano*[b], *Razieh M. Koushki* [a], *Majid Ahmadi* [a], *Enrique Ortí*[b], *Bart J. Kooi* [a], *Giuseppe Portale* [a], *Joaquín Calbo*[b], *Loredana Protesescu* [a,*]

[a]Zernike Institute for Advanced Materials, University of Groningen, Nijenborgh 4, Groningen, 9747AG, The Netherlands. E-mail: l.protesescu@rug.nll.protesescu@rug.nl

[b] Instituto de Ciencia Molecular, Universitat de València, c/ Catedrático José Beltrán, 2, 46980, Paterna, Spain

Table of Contents

	Description	Page
**I**	Theoretical calculations………………………………………..	4-10
**II**	Supporting Figures/Tables…………………………………..	11-21
**Figure S1**	X-Ray Diffraction (XRD), UV-Visible and PL spectroscopy of $[R\text{-}NH_3]_2PbBr_4$ prepared at low concentration (0.03 M) as per Protesescu *et al.* protocol[1] ……………………………….	11
**Figure S2**	SEM Images of 2D $[R\text{-}NH_3]_2SnBr_4$ RP Structure……………...	11
**Figure S3**	$CsSnI_3$ nanocrystal optical stability prepared at different concentrations…………………………………………………	12
**Figure S4**	XRD, UV-Visible and PL spectroscopy of $CsSnI_3$ NCs…………………………………………………..............	12
**Figure S5**	XRD of $PbBr_2$ (blue), $SnBr_2$ (green) and SnI_2 (wine) in OLA: DOPA with and without Cs injection ……………….………....	13
**Figure S6**	XRD of $SnBr_2$ (green) and SnI_2 (brown) in DOPA with and without Cs injection………………………………………...	14
**Figure S7**	STEM images of 6.8 nm and 10 nm $CsSnBr_3$ NCs at different magnifications with size histograms………………………..	15
**Table S1**	Elemental Analysis of 7 nm and 10 nm $CsSnBr_3$ NCs	16
**Figure S8**	Fourier Transform-Infrared (FT-IR) Spectroscopy of 7 nm and 10 nm $CsSnBr_3$ NCs	17
**Figure S9**	UV-Visible and PL spectroscopy of $CsSnBr_3$ NCs prepared at different Cs: Sn ratios……………………………….……………	18
**Figure S10**	XRD, UV-Visible and PL spectroscopy of $CsSnBr_3$ NCs prepared at different temperatures in ODE for Cs: Sn = 1: 3………………………………………………....................	19
**Figure S11**	SEM images for the reaction performed at 100ºC showing the co-existence of nanocubes with nanosheets…………………...	19
**Figure S12**	XRD, UV-Visible and PL spectroscopy of $CsSnBr_3$ NCs prepared at different temperatures in mesitylene for Cs: Sn =	

	1: 3…………………………………………………………….	20
**Figure S13**	Comparison of optical properties of 2D and 3D tin-halide perovskite materials.	21
References…………………………………………………………...……...		22

I. Theoretical Calculations

The minimum-energy structures of the 2D Ruddlesden-Popper phases ($L_2Cs_{n-1}M_nX_{3n+1}$, with n in the range 1-4) and those of the crystalline bulk $CsMX_3$ phases, where M stands for the metal cation (Pb^{2+}, Sn^{2+}), X for the anion (I^-, Br^-) and L for the OLA^+ ligand, were calculated under the Density Functional Theory (DFT) framework. Periodic boundary conditions were imposed and tier-2 numerical atom-centered orbitals (NAO) basis functions were used in conjunction with the GGA PBEsol functional,[2] as implemented in the FHI-aims code.[3] Relativistic effects were considered through the use of the scalar ZORA scheme. The initial structures for the bulk $CsMX_3$ phases were obtained from the Materials Project database,[4] considering γ-orthorhombic phases for $CsPbBr_3$ and $CsSnI_3$ (code references *mp-567629* and *mp-568570*, respectively) and the α-cubic phase for $CsSnBr_3$ (code reference *mp-27214*). The structural parameters of the minimum-energy crystal structures resulting from the full lattice and ionic optimization are listed in **Table S1**. The 2D Ruddlesden-Popper structures were constructed by hand from the optimized structures of the bulk phases and therefore α-cubic phase was assumed for $[R\text{-}NH_3]_2SnBr_4$ and γ-orthorhombic phases were constructed for $[R\text{-}NH_3]_2PbBr_4$ and $[R\text{-}NH_3]_2SnI_4$. The *k*-grid size was fixed for all systems at 6×6×6 due to the convergence of the unit cell energy and crystal lattice parameters, which are listed in **Table S2**. Large vacuum distances of at least 50 Å between the oleyl amine chains were set along the *c* axis to avoid clashes and interactions that could modify their arrangement.**Table S1**. Minimum-energy bulk $CsMX_3$ phase crystal lattice parameters obtained from the PBEsol/tier-2 NAO optimization.

Lattice Parameter	α-$CsSnBr_3$	γ-$CsPbBr_3$	γ-$CsSnI_3$
a (Å)	5.76	7.97	8.76
b (Å)	5.76	8.38	8.35
c (Å)	5.76	11.64	12.22
α (°)	90.0	90.0	90.0
β (°)	90.0	90.0	90.0
γ (°)	90.0	90.0	90.0

Table S2. Minimum-energy 2D Ruddlesden-Popper $[R\text{-}NH_3]_2MX_4$ phase crystal lattice parameters obtained from the PBEsol/tier-2 NAO optimization. Parameter *c* was set > 50 Å to avoid chain-to-chain interactions.

Lattice Parameter	$[R\text{-}NH_3]_2SnBr_4$	$[R\text{-}NH_3]_2PbBr_4$	$[R\text{-}NH_3]_2SnI_4$
a (Å)	5.68	7.94	8.44
b (Å)	5.67	8.05	8.39
c (Å)	-	-	-
α (°)	94.5	83.0	82.5
β (°)	93.9	94.9	94.9
γ (°)	88.8	91.1	90.9

The experimental evidences that the $[R\text{-}NH_3]_2MX_4$ phases could act as precursors for the bulk $CsMX_3$ structures, that structures of type $[R\text{-}NH_3]_2Cs_{n-1}M_nX_{3n+1}$ with $n \geq 1$ were detected via XRD measurements, and that the conversion to 3D NCs was more complete for the case of $CsSnBr_3$, prompted us to theoretically investigate the formation energies of the different phases and to devise the driving forces and interactions pointing to this behavior. The formation energies E_f for the $[R\text{-}NH_3]_2Cs_{n-1}M_nX_{3n+1}$ (or $(MX_2)_n(CsX)_{n-1}(OLAX)_2$) phases were evaluated as follows[5]

$$E_f = E(MX_2)_n(CsX)_{n-1}(OLAX)_2 - n\,E_{MX_2} - (n-1)\,E_{CsX} - 2\,E_{OLAX}$$

where all structures were optimized at the PBEsol/tier-2 NAO level of theory, considering periodic solid phases, and a *k*-grid size of 6×6×6. The initial structures for the optimization were retrieved from the Materials Project database, considering *Pnma* orthorhombic phases for $PbBr_2$ (*mp-28077*) and $SnBr_2$ (*mp-29862*), $C_{2/m}$ phase for SnI_2 (*mp-27194*) and cubic $Pm\bar{3}m$ phases for CsBr (*mp-22906*) and CsI (*mp-1056920*), as experimentally determined. The structures of OLABr and OLAI were constructed by example from that of the orthorhombic *Pbcm* phase of MAI (*mp-997570*). The lattice parameters for the $(OLA)_2Cs_{n-1}M_nX_{3n+1}$ phases (with $n > 1$) resulting from the PBEsol/tier-2 NAO optimization are listed in **Table S3**.

Table S3. 2D Ruddlesden-Popper $[R\text{-}NH_3]_2Cs_{n-1}M_nX_{3n+1}$phases (n > 1) crystal lattice parameters obtained from the PBEsol/tier-2 NAO optimization. Parameter *c* was set > 50 Å to avoid chain-to-chain interactions.

	$[R\text{-}NH_3]_2Cs_{n-1}Pb_nBr_{3n+1}$			$[R\text{-}NH_3]_2Cs_{n-1}Sn_nI_{3n+1}$			$[R\text{-}NH_3]_2Cs_{n-1}Sn_nBr_{3n+1}$		
	n =2	n = 3	n = 4	n = 2	n = 3	n = 4	n = 2	n = 3	n = 4
a (Å)	8.01	8.08	7.93	8.48	8.40	8.55	5.77	5.76	5.75
b (Å)	8.10	8.16	8.26	8.52	8.67	8.49	5.72	5.72	5.73
c (Å)	-	-	-	-	-	-	-	-	-

α (°)	94.1	106.0	91.3	84.8	101.5	86.4	78.1	91.7	88.9
β (°)	84.5	90.3	89.7	82.2	89.6	87.3	89.9	83.1	94.5
γ (°)	90.0	90.2	90.0	89.8	90.0	90.1	89.9	90.1	90.0

The values of the $[R\text{-}NH_3]_2Cs_{n-1}M_nX_{3n+1}$ formation energies are listed in **Table S4**. From them, it can be concluded that the formation of the $[R\text{-}NH_3]_2MX_4$ phase is in all cases favorable, as negative values were predicted for the three systems. Furthermore, the formation energies of $[R\text{-}NH_3]_2Cs_{n-1}M_nX_{3n+1}$ become more negative as the value of *n* increases. These two results agree with the isolation of the $[R\text{-}NH_3]_2MX_4$ phases and the co-existence of higher-order *n* structures depending on the experimental conditions. The formation energy in the case of $[R\text{-}NH_3]_2SnBr_4$ is significantly smaller than that for $[R\text{-}NH_3]_2PbBr_4$ and $[R\text{-}NH_3]_2SnI_4$. Moreover, the net stabilization of each of the higher-order *n* structures (n = 4) compared to the $[R\text{-}NH_3]_2MX_4$ phase is larger for the Sn-Br combination (0.79 eV), in comparison with that obtained for Sn-I (0.50 eV) and Pb-Br (0.68 eV). These results point towards the more complete formation of the 3D bulk $CsSnBr_3$ phase, as well as for higher-order $[R\text{-}NH_3]_2Cs_{n-1}M_nX_{3n+1}$ structures. For comparison, the formation energies of the $[R\text{-}NH_3]_2Cs_{n-1}M_nX_{3n+1}$ phases with n = 4 were considered as those for the 3D bulk structures.

Table S4. PBEsol/tier-2 NAO formation energies per formula unit (E_f in eV) calculated for the $[R\text{-}NH_3]_2Cs_{n-1}M_nX_{3n+1}$ phases.

	n = 1	n = 2	n = 3	n = 4 ("bulk")
$(OLA)_2Cs_{n-1}Pb_nBr_{3n+1}$	-0.247	-0.440	-0.588	-0.928
$(OLA)_2Cs_{n-1}Sn_nBr_{3n+1}$	-0.175	-0.426	-0.643	-0.970
$(OLA)_2Cs_{n-1}Sn_nI_{3n+1}$	-0.325	-0.442	-0.569	-0.822

To disentangle the trends observed both experimentally and by means of DFT calculations, the interactions between the oleyl amine chains within the $[R\text{-}NH_3]_2MX_4$ structures were investigated through the calculation of the non-covalent index (NCI).[6] This magnitude is based on the reduced density gradient *s* and the electron density *p* according to the following equation

$$s = \frac{|\nabla\rho|}{2(3\pi^2)^{1/3}\rho^{4/3}}$$

In the presence of intermolecular interactions there is a shift in the reduced density, pointing to the creation of critical interacting points between atoms or molecular fragments that can be visualized (see **Figure 2a**). Furthermore, the value of the density can be used to assign the strength of the interaction, while the sign of the Laplacian of the density $\nabla^2\rho$ (λ_2) can be used to distinguish between repulsive interactions ($\lambda_2 > 0$) and those with van der Waals character ($\lambda_2 \lesssim 0$). The NCIPLOT code was used on top of a 3x3x1 supercell of the different $[R\text{-}NH_3]_2MX_4$ systems,[7] resulting from the DFT optimization, but removing the MX_6 octahedra. Promolecular densities were used as they provide a reasonable ratio between the computational cost and the accuracy of the calculations. The magnitude of interaction between the OLA^+ cations and the perovskite MX_6 octahedra is crucial for the desorption of the cations, required to form more complex structures of increased dimensionality. To estimate the OLA^+-perovskite interaction, 3x3x1 supercells were constructed from the PBEsol/tier-2 NAO minimum-energy structures of $[R\text{-}NH_3]_2MX_4$ and removing the central cation. As the terminal OLA^+ mainly interacts with the perovskite through the ammonium group, the oleyl amine chains were substituted by shorter cations with five C atoms to reduce the computational cost. The interaction energy E_i was then calculated at the PBEsol/tier-2 NAO basis set level of theory as follows

$$E_i = E_{bulk} - E_{cation} - E_{desorb}$$

where E_{bulk} corresponds to the energy of the 3x3x1 supercell with short cations, E_{cation} is the energy of the desorbed short cation, and E_{desorb} is the energy of the 3x3x1 supercell once the central short cation was desorbed. All the calculations were performed with periodic boundary conditions. The results, which are listed in **Table S5**, were calculated accounting for intermolecular interactions with the use of the Tkatchenko-Scheffler dispersion correction as available in the FHI-aims code.[8] Irrespective of the use of the dispersion correction, the interaction energy is lower for the $[R\text{-}NH_3]_2SnBr_4$ structure, which ensures a weaker oleyl amine-perovskite octahedra interaction. This result, combined with the more destabilizing interaction between oleyl amine chains, favors the cation desorption required to form bulkier phases in the case of $[R\text{-}NH_3]_2SnBr_4$.

Table S5. Oleyl amine-perovskite interaction energy (in eV) calculated with and without dispersion correction at the PBEsol/tier-2 NAO basis set level of theory.

	no correction	dispersion-corrected
$[R\text{-}NH_3]_2PbBr_4$	−5.91	−6.39
$[R\text{-}NH_3]_2SnBr_4$	−4.93	−5.40
$[R\text{-}NH_3]_2SnI_4$	−5.31	−5.76

The interlayer distance (d-spacing) for the $[R\text{-}NH_3]_2MX_4$ materials was also inspected by computational simulations to devise the degree of chain intercalation that could correspond to the experimental values shown by XRD measurements in the range of 3.8–4.2 nm. To inspect the effect of temperature and dynamic disorder in the d-spacing, classical Molecular Dynamics simulations were performed on top of a 3x3x1 supercell of $[R\text{-}NH_3]_2SnBr_4$, as a representative example, with complete oleyl amine chains but without the perovskite octahedra (see **Figure 2b**).

The system was parametrized according to the CHARMM force field using CGenFF through the CHARMM-GUI interface.[9] The DFT lattice parameters from the optimization were used to impose periodic boundary conditions and the N atoms were frozen during the trajectories. First, 1000 steps of classical minimization were performed keeping the unit cell fixed. Then, a NVT thermalization trajectory of 50 ns with a time step of 2 fs was used to ensure that the system was correctly equilibrated and thermalized. A significant contraction of the oleylamine chains along the *c* axis occurred and therefore several NVT trajectories were performed, adjusting the size of the unit cell, until no further contraction was observed. The d-spacing value was estimated as the distance between the planes generated by the cation N atoms plus an average distance of 2.25 Å between the N and the plane that crosses the center of the perovskite octahedra. Even though our simulations neglect the effect of the MX_6 octahedra and that of the displacement of the chains on the final arrangement, our results show that large intercalation of the chains is not required to attain the short d-spacings obtained experimentally.

II. Supporting Figures

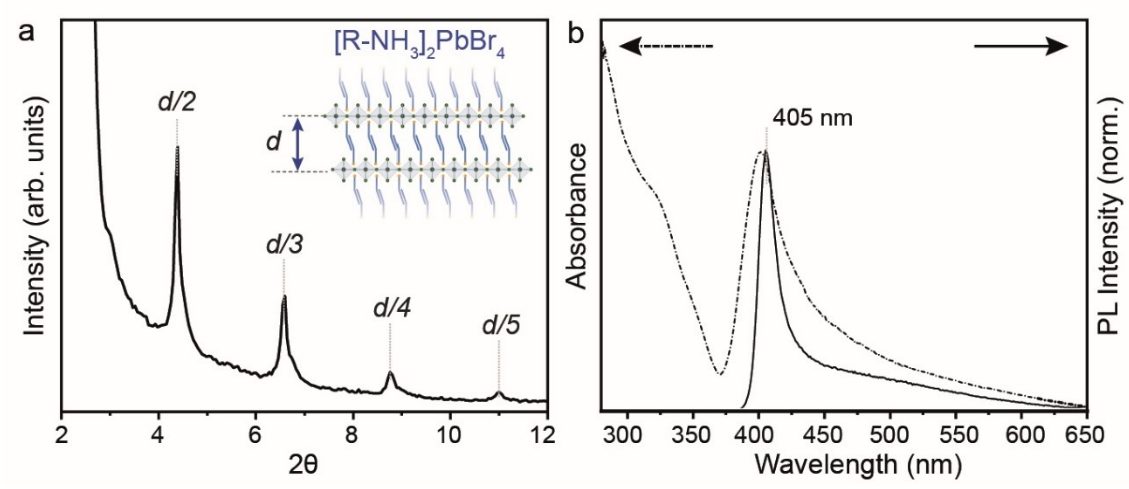

Figure S1. *(a) XRD pattern of 2D [R-NH_3]$_2$$PbBr_4$ RP structure prepared at low concentration (0.03 M) as per Protesescu et al. protocol*[1]. *(b) UV-visible and PL spectroscopy of 2D [R-NH_3]$_2$$PbBr_4$ RP structure.*

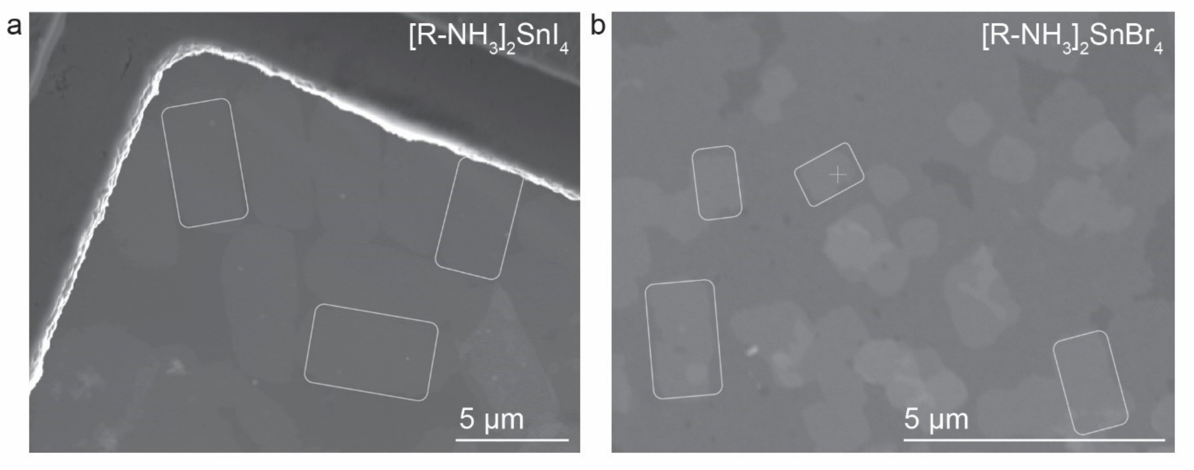

Figure S2. *(a) and (b) SEM images of 2D [R-NH_3]$_2$$SnI_4$ and [R-NH_3]$_2$$SnBr_4$ RP structure showing nanosheet formation with the aspect ratio of 3:2 shown via rounded rectangles respectively.*

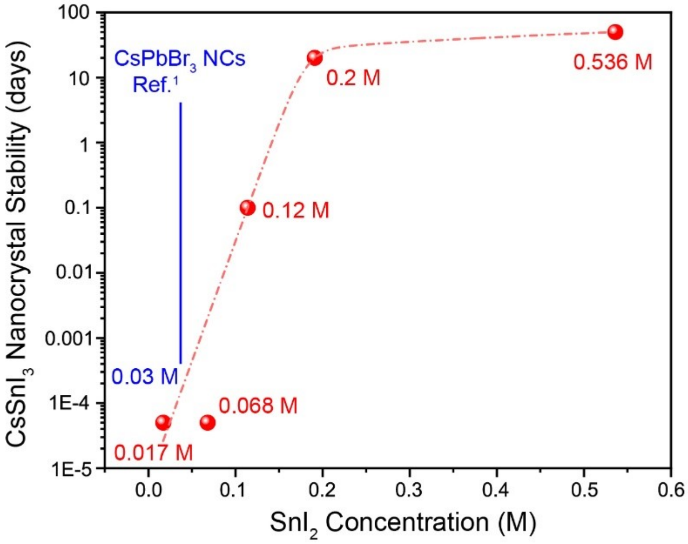

***Figure S3.**. The stability of $CsSnX_3$* NCs *(in days) is plotted with SnI_2 concentration (M) in the reaction mixture with marked in blue the concentration that works well for Pb-halide perovskite NCs as reported by Protesescu et al.*[1]

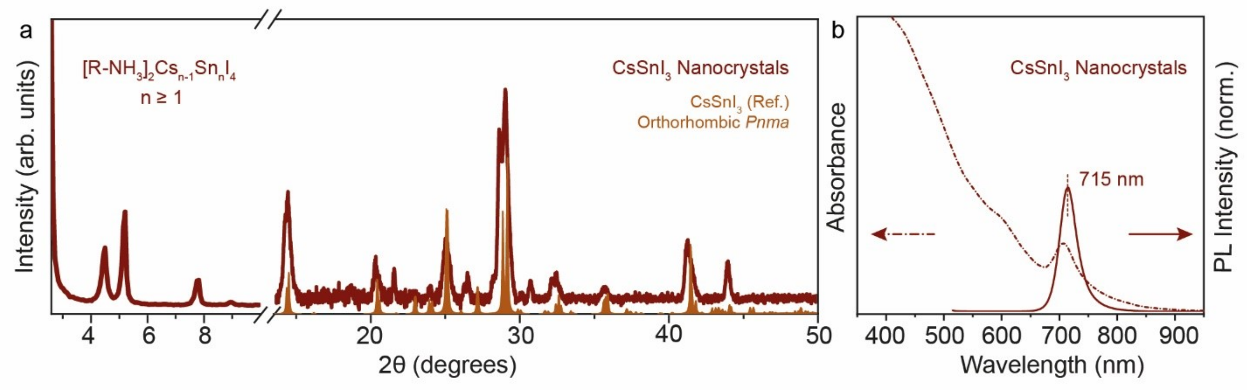

***Figure S4**. (a) XRD at low and high 2θ angles for $CsSnI_3$ NCs in comparison with orthorhombic Pnma crystal phase reference*[11]*. (b) UV-visible and photoluminescence spectroscopy of $CsSnI_3$ NCs with PL peak maxima at 715 nm.*

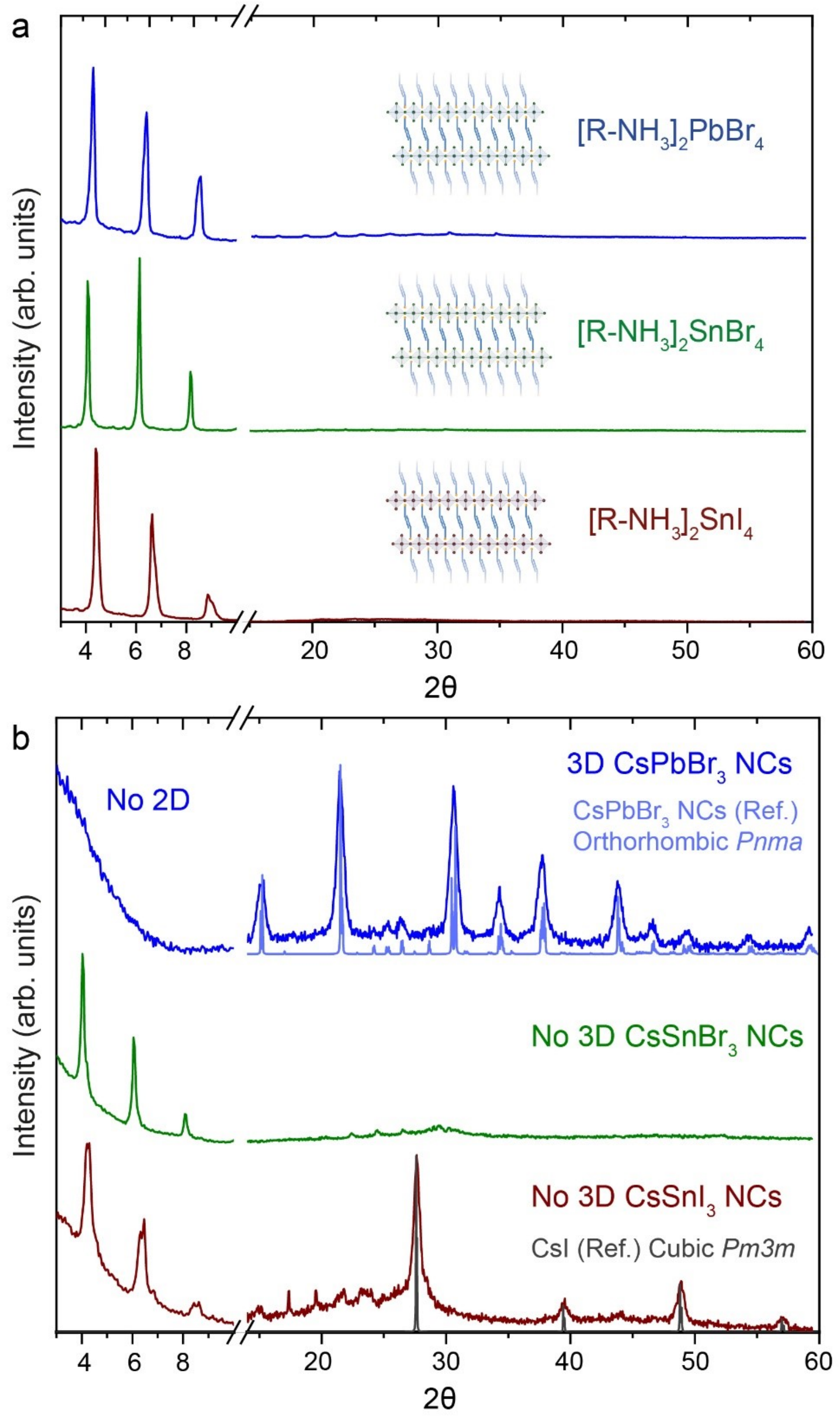

Figure S5. *(a) XRD of 2D $[R\text{-}NH_3]_2MX_4$ RP structures ($[R\text{-}NH_3]_2PbBr_4$ – blue, $[R\text{-}NH_3]_2SnBr_4$ – green, $[R\text{-}NH_3]_2SnI_4$ – brown) synthesized with OLA:DOPA = 1:1. (b) XRD of Cs injection in attempt to form 3D $CsMX_3$* NCs *($CsPbBr_3$ – blue, ($CsSnBr_3$ – green, $CsSnI_3$ – brown) synthesized with OLA:DOPA = 1:1.*

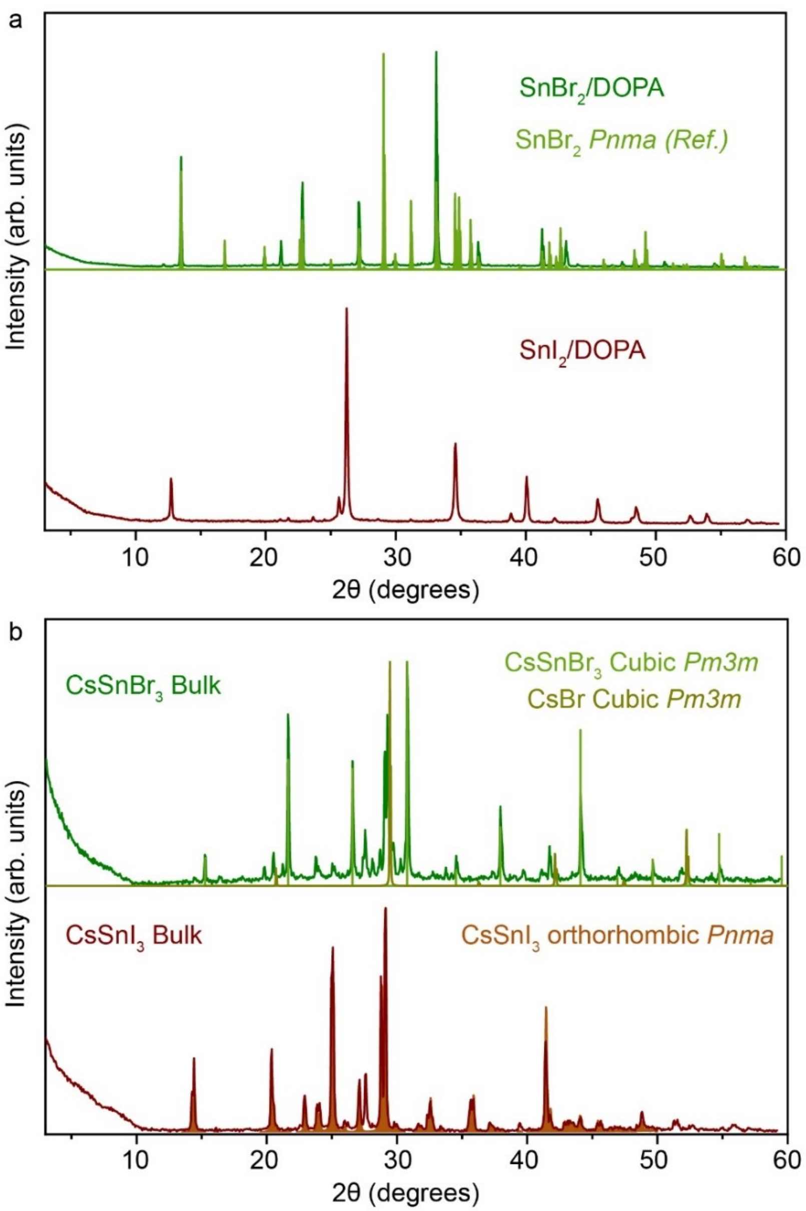

Figure S6. *(a)* XRD *of SnX_2 halide salts in DOPA quenched to room temperature without Cs injection. The $SnBr_2$ Pnma reference is reproduced from Eckold et al.*[10] *(b)* XRD *of SnX_2 halide salts in DOPA quenched to room temperature with Cs injection producing bulk Sn perovskites. The bulk references are reproduced from cited literature.*[11-13]

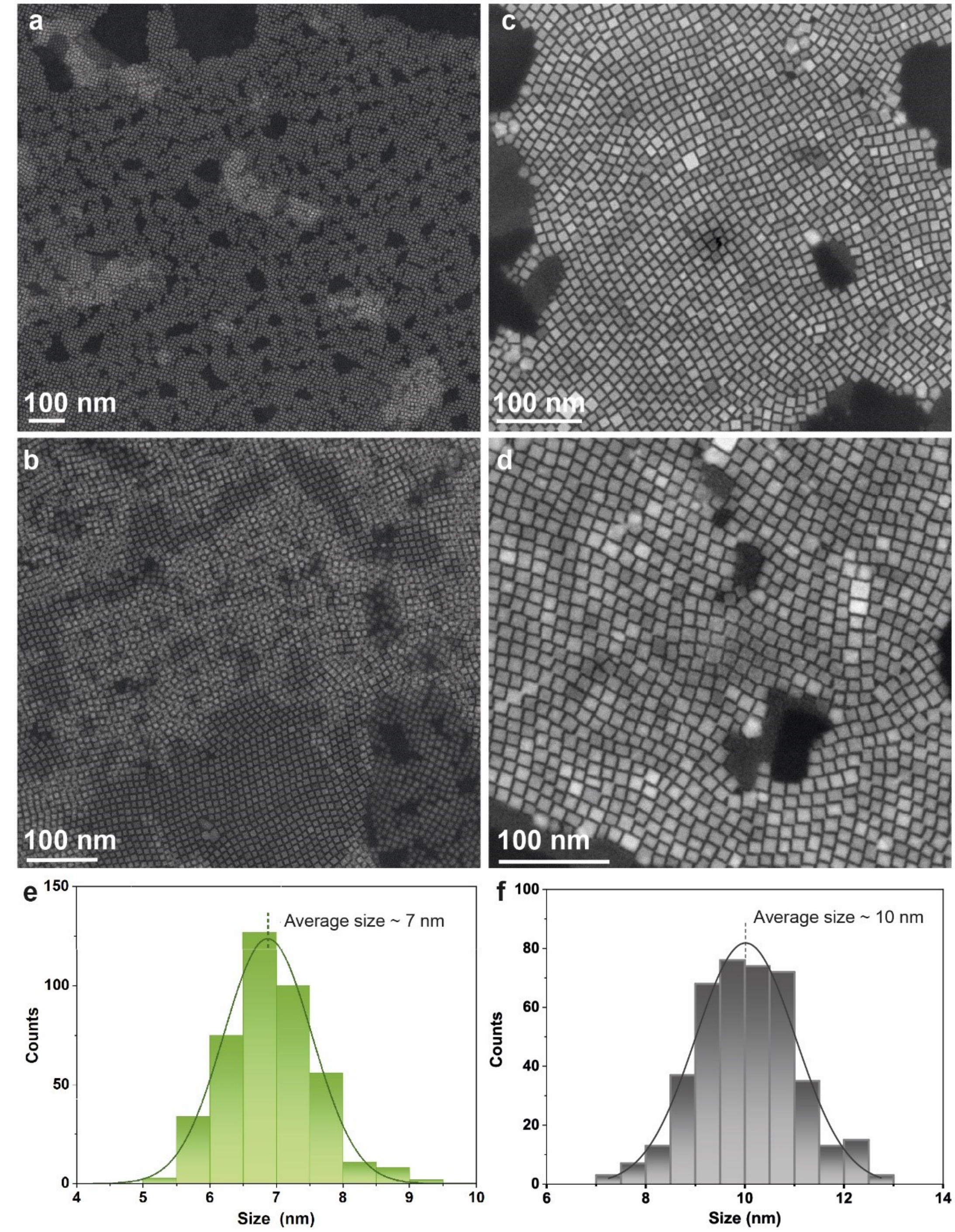

Figure S7.*(a) and (b) STEM images of 7 nm $CsSnBr_3$ NCs at different magnifications. (c) Size histogram showing the average edge size to be 7 nm. (e) and (f) STEM images of 10 nm $CsSnBr_3$ NCs at different magnifications. (f) Size histogram showing the average edge size to be 10 nm.*

Table S1. Elemental Analysis

STEM-EDX *(norm. to Sn atm. fraction)*			
Sample	Cs (atm. fraction)	Sn (atm. fraction)	Br (atm. fraction)
7 nm $CsSnBr_3$ NCs	0.7	1.0	2.7
10 nm $CsSnBr_3$ NCs	0.8	1.0	2.3
ICP-MS *(norm. to Sn at. fraction)*			
Sample	Cs (atm. fraction)	Sn (atm. fraction)	Br (atm. fraction)
7 nm $CsSnBr_3$ NCs	-	1.0	3.2
10 nm $CsSnBr_3$ NCs	-	1.0	1.8

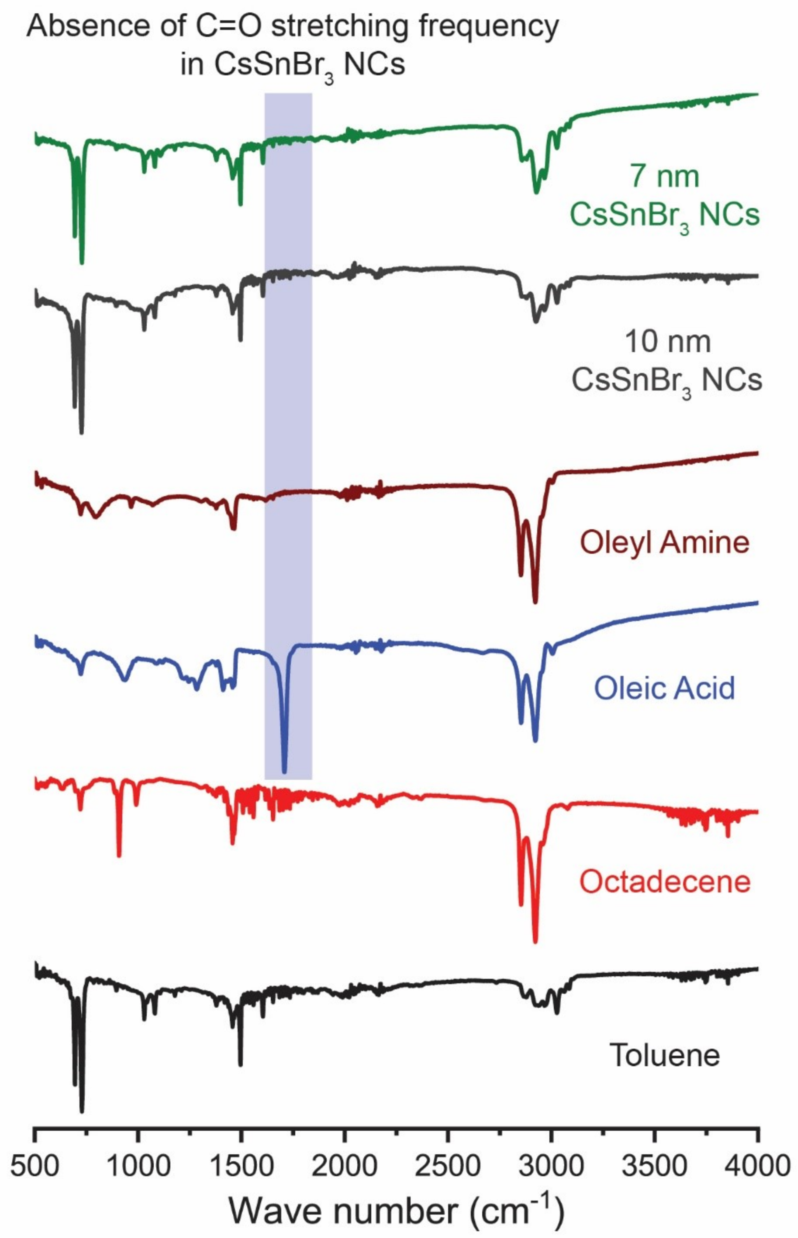

Figure S8. *Fourier Transform-Infrared (FT-IR) Spectroscopy of 7 nm (green) and 10 nm (dark grey) $CsSnBr_3$ NCs in comparison with oleic acid, oleyl amine, octadecene, and toluene, showing the disappearance of C=O stretching frequency from free ligands.*

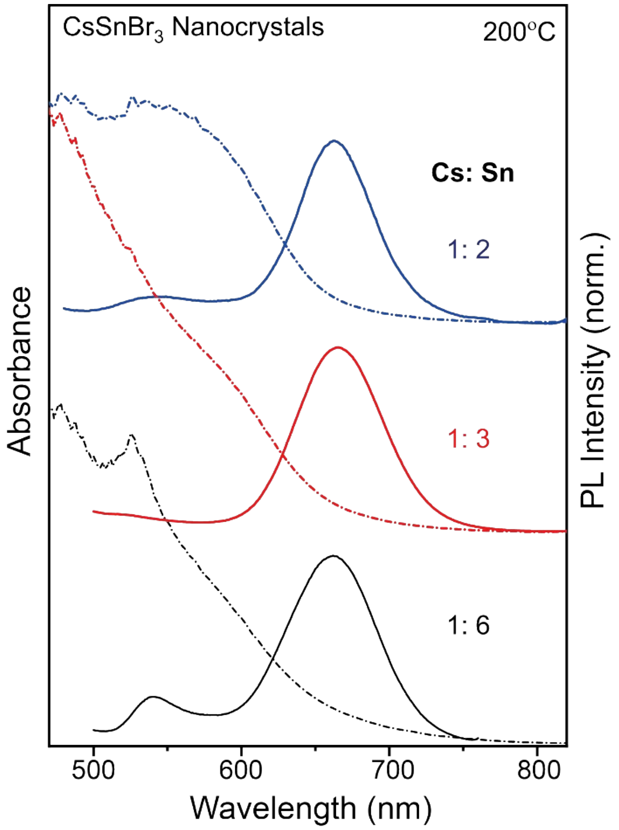

Figure S9. *UV-Visible and PL spectroscopy of $CsSnBr_3$ NCs synthesized with different cation rations of Cs: Sn at 200°C.*

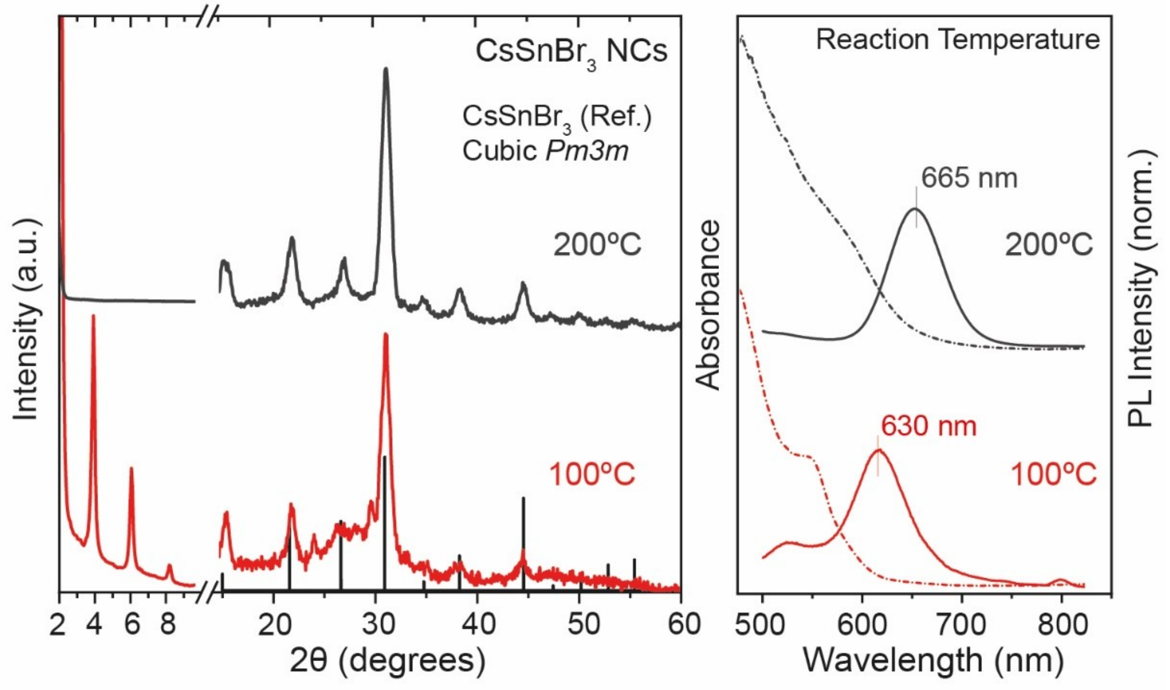

Figure S10. *(a)* XRD *of $CsSnBr_3$ NCs synthesized in ODE at 200ºC (grey) and 100ºC (red) plotted with $CsSnBr_3$ cubic Pm3m bulk reference[12] showing the co-existence of 2D and 3D perovskite structures at low temperature injection. (b) UV-Visible and PL spectroscopy of $CsSnBr_3$ NCs in ODE at different temperatures.*

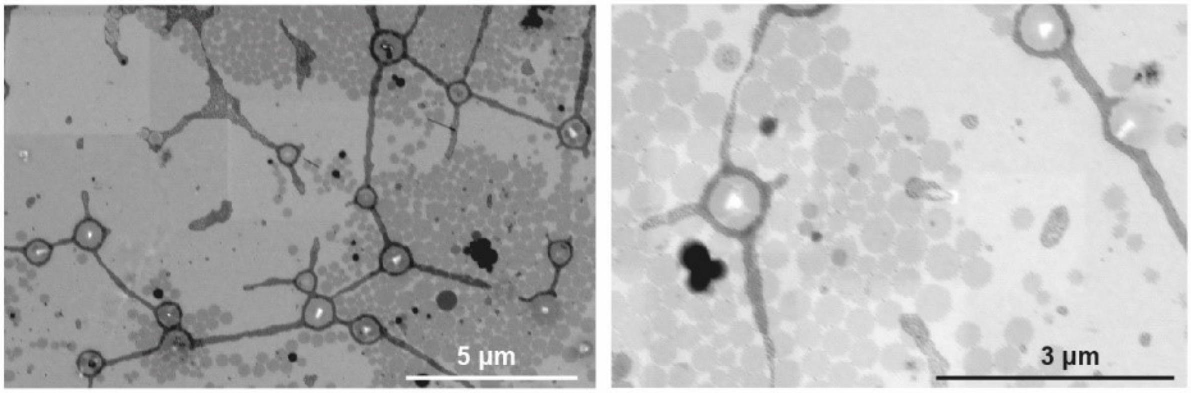

Figure S11*. SEM Images showing the co-existence of 3D $CsSnBr_3$ NCs and 2D nanosheets when the reaction is performed at 100ºC.*

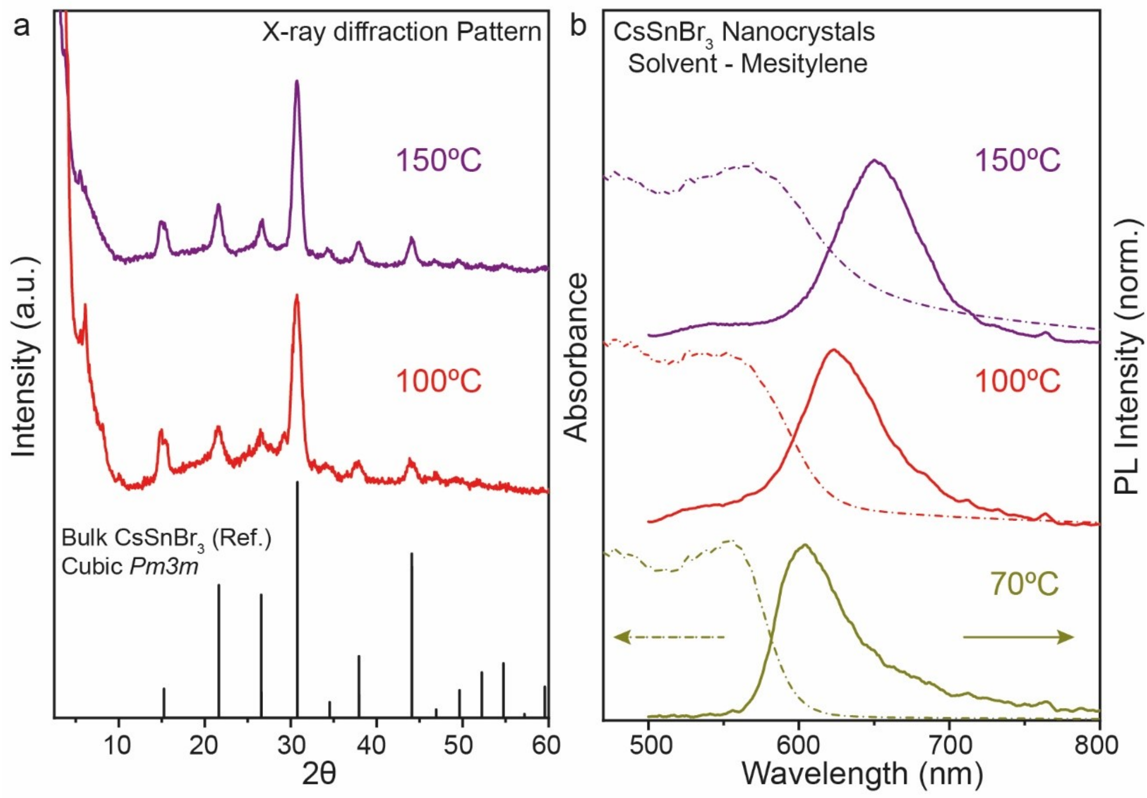

***Figure S12**. (a)* XRD *of $CsSnBr_3$ NCs synthesized in mesitylene at 150ºC and 100ºC plotted with $CsSnBr_3$ cubic Pm3m bulk reference[12]. (b) UV-Visible and PL spectroscopy of $CsSnBr_3$ NCs in mesitylene at different temperatures.*

***Figure S13**. Comparison of optical properties of 2D and 3D tin-halide perovskite materials.*

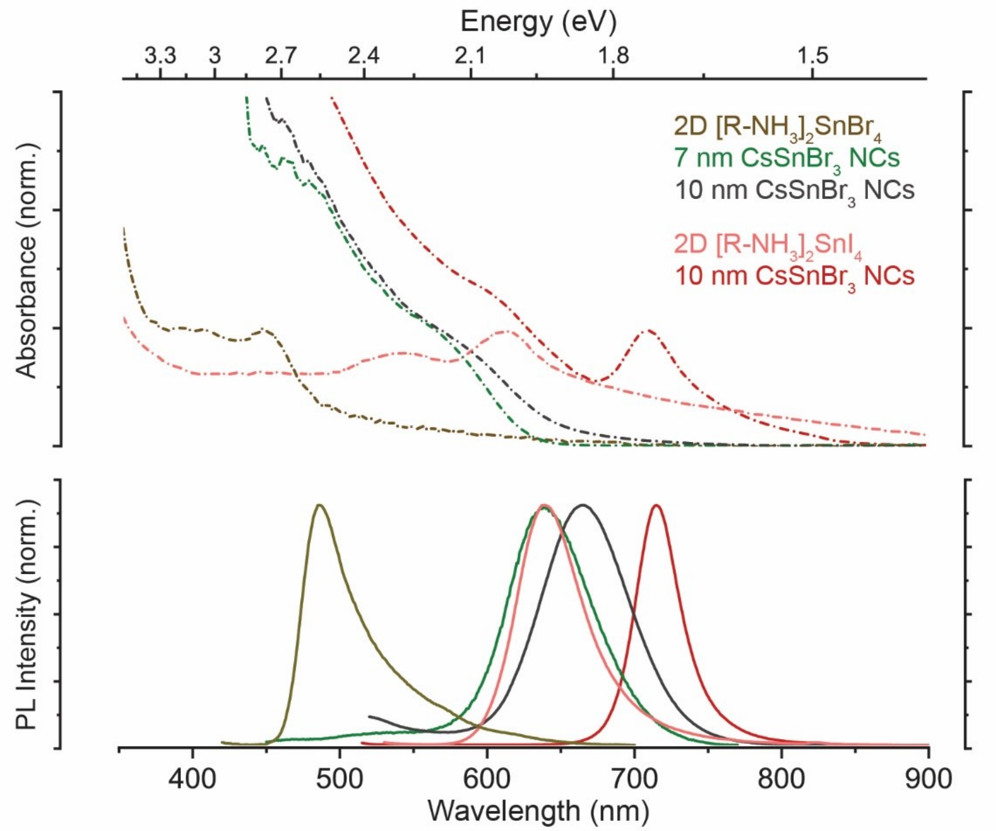

References:

(1) Protesescu, L.; Yakunin, S.; Bodnarchuk, M. I.; Krieg, F.; Caputo, R.; Hendon, C. H.; Yang, R. X.; Walsh, A.; Kovalenko, M. V. Nanocrystals of Cesium Lead Halide Perovskites (CsPbX3, X = Cl, Br, and I): Novel Optoelectronic Materials Showing Bright Emission with Wide Color Gamut. *Nano Lett.* **2015**, *15* (6), 3692-3696.

(2) Perdew, J. P.; Ruzsinszky, A.; Csonka, G. I.; Vydrov, O. A.; Scuseria, G. E.; Constantin, L. A.; Zhou, X.; Burke, K. Restoring the Density-Gradient Expansion for Exchange in Solids and Surfaces. *Phys. Rev. Lett.* **2008**, *100* (13), 136406.

(3) Blum, V.; Gehrke, R.; Hanke, F.; Havu, P.; Havu, V.; Ren, X.; Reuter, K.; Scheffler, M. Ab initio molecular simulations with numeric atom-centered orbitals. *Comput. Phys. Commun.* **2009**, *180* (11), 2175-2196.

(4) Jain, A.; Ong, S. P.; Hautier, G.; Chen, W.; Richards, W. D.; Dacek, S.; Cholia, S.; Gunter, D.; Skinner, D.; Ceder, G.; et al. Commentary: The Materials Project: A materials genome approach to accelerating materials innovation. *APL Mater.* **2013**, *1* (1), 011002.

(5) Quan, L. N.; Yuan, M.; Comin, R.; Voznyy, O.; Beauregard, E. M.; Hoogland, S.; Buin, A.; Kirmani, A. R.; Zhao, K.; Amassian, A.; et al. Ligand-Stabilized Reduced-Dimensionality Perovskites. *J. Am. Chem. Soc.* **2016**, *138* (8), 2649-2655.

(6) Johnson, E. R.; Keinan, S.; Mori-Sánchez, P.; Contreras-García, J.; Cohen, A. J.; Yang, W. Revealing Noncovalent Interactions. *J. Am. Chem. Soc.* **2010**, *132* (18), 6498-6506.

(7) Contreras-García, J.; Johnson, E. R.; Keinan, S.; Chaudret, R.; Piquemal, J.-P.; Beratan, D. N.; Yang, W. NCIPLOT: A Program for Plotting Noncovalent Interaction Regions. *J. Chem. Theory Comput.* **2011**, *7* (3), 625-632.

(8) Tkatchenko, A.; Scheffler, M. Accurate Molecular Van Der Waals Interactions from Ground-State Electron Density and Free-Atom Reference Data. *Phys. Rev. Lett.* **2009**, *102* (7), 073005.

(9) Jo, S.; Kim, T.; Iyer, V. G.; Im, W. CHARMM-GUI: A web-based graphical user interface for CHARMM. *J. Comput. Chem.* **2008**, *29* (11), 1859-1865.

(10) Eckold, P.; Hügel, W.; Dinnebier, R. E.; Niewa, R. Two Modifications of Tin(II) Bromide. *Z. Anorg. allg. Chem.* **2015**, *641* (8-9), 1467-1472.

(11) Chung, I.; Song, J.-H.; Im, J.; Androulakis, J.; Malliakas, C. D.; Li, H.; Freeman, A. J.; Kenney, J. T.; Kanatzidis, M. G. CsSnI3: Semiconductor or Metal? High Electrical Conductivity and Strong Near-Infrared Photoluminescence from a Single Material. High Hole Mobility and Phase-Transitions. *J. Am. Chem. Soc.* **2012**, *134* (20), 8579-8587.
(12) Fabini, D. H.; Laurita, G.; Bechtel, J. S.; Stoumpos, C. C.; Evans, H. A.; Kontos, A. G.; Raptis, Y. S.; Falaras, P.; Van der Ven, A.; Kanatzidis, M. G.; et al. Dynamic Stereochemical Activity of the Sn2+ Lone Pair in Perovskite CsSnBr3. *J. Am. Chem. Soc.* **2016**, *138* (36), 11820-11832.
(13) Wyckoff, R. W. G. *Crystal Structures - Volume 1*; Interscience Publishers New York, 1963.
